# Physiology-based toxicokinetic modelling of aluminium in rat and man

**DOI:** 10.1007/s00204-021-03107-y

**Published:** 2021-08-14

**Authors:** Christoph Hethey, Niklas Hartung, Gaby Wangorsch, Karin Weisser, Wilhelm Huisinga

**Affiliations:** 1 Junior Research Group Toxicokinetic Modelling, Department Exposure, German Federal Institute for Risk Assessment, Berlin, Germany; 2 Institute of Mathematics, Mathematical Modelling and Systems Biology, University of Potsdam, Potsdam, Germany; 3 Paul-Ehrlich-Institut (Federal Institute for Vaccines and Biomedicines), Langen, Germany

**Keywords:** PBTK, Toxicokinetics, ^26^Al, Aluminium

## Abstract

A sufficient quantitative understanding of aluminium (Al) toxicokinetics (TK) in man is still lacking, although highly desirable for risk assessment of Al exposure. Baseline exposure and the risk of contamination severely limit the feasibility of TK studies administering the naturally occurring isotope ^27^Al, both in animals and man. These limitations are absent in studies with ^26^Al as a tracer, but tissue data are limited to animal studies. A TK model capable of inter-species translation to make valid predictions of Al levels in humans—especially in toxicological relevant tissues like bone and brain—is urgently needed. Here, we present: (i) a curated dataset which comprises all eligible studies with single doses of ^26^Al tracer administered as citrate or chloride salts orally and/or intravenously to rats and humans, including ultra-long-term kinetic profiles for plasma, blood, liver, spleen, muscle, bone, brain, kidney, and urine up to 150 weeks; and (ii) the development of a physiology-based (PB) model for Al TK after intravenous and oral administration of aqueous Al citrate and Al chloride solutions in rats and humans. Based on the comprehensive curated ^26^Al dataset, we estimated substance-dependent parameters within a non-linear mixed-effect modelling context. The model fitted the heterogeneous ^26^Al data very well and was successfully validated against datasets in rats and humans. The presented PBTK model for Al, based on the most extensive and diverse dataset of Al exposure to date, constitutes a major advancement in the field, thereby paving the way towards a more quantitative risk assessment in humans.

**Supplementary Information:**

The online version contains supplementary material available at 10.1007/s00204-021-03107-y.

## Introduction

Aluminium (Al), as an ubiquitous element, is continuously absorbed from food and drinking water, resulting in baseline levels of Al in body fluids and tissues, while lacking any known physiological function (Snyder et al. 1975; Yokel et al. 2001a, b). Sources of additional short-term exposure are medicinal products containing Al, such as antacids, haemodialysis fluids, intravenous feeding solutions, vaccines, or subcutaneous immunotherapeutics. Additional Al exposure results from food supplements and cosmetics (Tietz et al. 2019). As Al is known to be potentially toxic for the central nervous system and bone, knowledge of its toxicokinetics (TK) is crucial to evaluate the risk associated with intended or unintended exposure.

The most recent assessment of aluminium (Al) exposure for the general population including food, water, cosmetics, and medicinal products (Tietz et al. 2019) indicates that the corresponding tolerable weekly intake (TWI) (EFSA 2008) and even the provisional tolerable weekly intake (PTWI) (WHO 2011) may be exceeded for certain populations. To analyse, understand, and ultimately control the sources of exposure, a proper understanding of the functional link between external and internal exposure is a necessary requirement. In this regard, toxicokinetic (TK) models are ideally suited to predict the internal exposure in man in terms of blood concentrations, tissue distribution, and also excretion rates of Al for defined external exposure scenarios. Although several attempts have been made in the past to fit individual study data [reviewed by Priest (2004) and discussed in Weisser et al. (2017)], a validated TK model based on an extensive and diverse dataset of Al exposure suitable for simulations in animals and humans still constitutes a ‘scientific gap’ (Krewski et al. 2007).

The development of a TK model relies on the availability of data from intervention studies, where animals and humans are exposed to Al via different routes of exposure. There is a vast amount of reports on studies using the naturally and ubiquitously occurring isotope ^27^Al. The design of ^27^Al studies, however, is limited due to existing baseline levels and risk of sample contamination. This severely restricts the observation time span and the ability to quantify Al distribution in tissues, e.g., in animal studies. Therefore, regarding long-term TK and tissue distribution, the most valuable data on Al TK are from studies using the stable ^26^Al isotope as a tracer. We recently reviewed the corresponding literature on this type of data (Weisser et al. 2017). Differences in study designs, reported outcomes, and in particular the lack of a curated dataset on ^26^Al study data, however, limited so far the use of this very valuable source of information. To integrate data across different species, physiology-based (PB)TK models represent the most suitable approach for the inter-species translation.

The objectives of this article are thus (i) the compilation of a comprehensive *curated* ^26^Al kinetic dataset; and (ii) the development of a PBTK model for Al. Compiling data from multiple studies requires careful inspection of the study protocols and a clear definition of inclusion and exclusion criteria. Overly restrictive definitions result in a small, homogenous, and highly standardised dataset, with potentially limited translatability. Overly lax definitions result in a larger, more heterogeneous, yet poorly standardised dataset that might require including dependences of covariates and features of Al kinetics that are currently not sufficiently well understood to be included mechanistically into the PBTK model. To cope with the high diversity in reported outcomes in terms of units [e.g., concentration in g/L, fraction of ingested dose (fid), fid/g tissue, etc.], we used a PB scaling approach based on the reported study designs. During the curation process, we identified inconsistent data points as well as study data that were published multiple times in different publications without explicit reference. The resulting comprehensive and curated dataset allows for the first time to analyse ^26^Al kinetics beyond particular features of individual studies.

To account for the already visually present large inter-study and inter-individual variability, we implemented the PBTK model into a non-linear mixed-effect modelling context (e.g., Lavielle and Bleakley 2015), using the curated ^26^Al dataset as training data for parameter estimation. This statistical framework allows us to rigorously differentiate between inter-individual/inter-study variability and uncertainty, and to propagate variability and uncertainty to the observation level for an assessment of model predictivity. After parameter estimation, the PBTK model was evaluated against different validation datasets, comprising recently obtained ^27^Al plasma data in rats over 24 h (Weisser et al. 2019), ^26^Al full-body retention data in humans over 375 weeks (Newton and Talbot 2012), and recent ^26^Al blood data in humans over 3 weeks (de Ligt et al. 2018).

## Methods

### Literature search

The literature was screened for reports on ^26^Al studies based on a Google Scholar search for the terms ‘26Al OR Al-26’ and in PubMed for ‘26Al AND PK’ as of December 2015. In Weisser et al. (2017, Tables 1 and 2), the identified human and animal studies are reported together with the most relevant citations (12 references for human studies and 30 references for animal studies; with some references reporting about multiple studies). In addition, further references were identified that also report about aspects of the already identified studies, including diploma and Ph.D. theses or follow-up publications, etc. These, however, were not systematically reported in Weisser et al. (2017). As of December 2019, we repeated the 2015 search. The updated 2019 query identified two additional reports (de Ligt et al. 2018; Jugdaohsingh et al. 2000).

### Data extraction from references

If available, we extracted numerical values from tables and the text from the original publication. If not available, we used the free software WebPlotDigitizer (Rohatgi 2015) to digitise the kinetic data from figures. The quality of graphical representations is not always well suited for extracting the underlying numerical values via digitizing tools. As a consequence, we excluded non-plausible sampling time-points $< {1}\hbox { s}$ post-administration. All extracted data from figures were double-checked by re-plotting and comparison to the figures published in the original study. In addition, following the 4-eyes-principle, we ensured completeness and correctness during the curation process.

As tissue sampling typically requires to sacrifice the animal, observations at multiple time-points usually represent multiple individual animals. Due to lack of knowledge, we assumed the same to hold for blood or plasma sampling in animals. Thus, data from animal studies were reported per study. In contrast to animal studies, human studies report the data per individual. Instead of grouping several human individuals together, the knowledge on repeated measurements per individual was kept in the data. Consequently, for animals, we may only characterise inter-study variability (due to lack of repeated measurements in individual rats), while for humans, we may also capture inter-individual variability in addition to inter-study variability.

To uniquely refer to a series of observations (either from an animal study or a human individual), each observation was associated with a summary identifier following the scheme:

$$\begin{aligned} \hbox {[Study]-[Salt]-[Administration]-[Comment]-[Species]-[Body Weight]}. \end{aligned}$$

The identifiers included study publication (first author and year), the administered salt in terms of aqueous solutions of chloride (AlChl) and citrate salt (AlCit), and the route of administration (orally = po, or intravenously = iv). Additional treatments, such as co-administration of citrate (addcit), non-fed state (fasted), or special water supply (hardwater or softwater), were included in the comment field. Finally, the species and body weight (BW) were included.

For data handling and plotting, we used Matlab 2015a (8.5.0.197613) by MathWorks. With respect to tissue nomenclature, we treated serum and plasma equally. Likewise, we made no distinction between samples from brain and grey matter.

### De-aggregation of summary data

Some studies only report summary data in form of number of measurements $N>1$, mean *m*, and standard deviation sd instead of the individual observations. For data analyses, these summary data cannot be treated the same way as individual observations. To be able to use summary data jointly with single measurements, we ‘de-aggregated’ the summary data: to this end, we generated *N* samples $X_1,\ldots ,X_N$ assuming a log-normal distribution with the reported mean *m* and standard deviation sd. More precisely, we sampled on the log scale from a normal distribution $\mathcal {N}(\mu ,\sigma ^2)$ with

1 $$\begin{aligned} \sigma ^2 = \log \left( \frac{\mathrm{sd}^2}{m^2} +1 \right) \quad \text {and}\quad \mu = \log \left( m \right) - \frac{\sigma ^2}{2}, \end{aligned}$$

and scaled, centred, and finally transformed the data back to the original scale via

2 $$\begin{aligned} Y_k = \exp \left( X_k \times \frac{\sqrt{\sigma ^2}}{\mathrm{sd}(X)} - m(X) + \mu \right) , \end{aligned}$$

for $k=1,\ldots ,N$ with sample mean *m*(*X*) and standard deviation sd(*X*), respectively.

### Conversion to the common base units

For ease of comparison, we transformed all mass concentrations to g/L. In view of the aim to compile a comprehensive dataset for PBTK model development, we further converted all units to the (most abundant) common unit ‘fraction of ingested dose’ (fid). This necessitated conversion of concentration units per g or L tissue. Typically, however, blood volume or tissue weights were not reported jointly with the corresponding measurements. Due to lack of these data, we predicted the missing physiological parameter value required for unit conversions (blood/plasma volumes and tissue weights/volumes) for rats and humans using a scaling approach that proved successful in PBPK modelling. It is based on reference weights/volumes stratified for sex and age, and allometric scaling using BW for rats or linear scaling using BW, lean BW (LBW), and body height (BH) for humans (Huisinga et al. 2012).

In addition, glomerular filtration rate (GFR) for rats was allometrically scaled according to $\mathrm {GFR}{} = \mathrm {SF}_\mathrm {unit} \times \mathrm {GFR}_\mathrm {ref}\times (\mathrm {BW}/\mathrm {BW}_\mathrm {ref})^{3/4}$ with $\mathrm {GFR}_\mathrm {ref}= {1.31}\,\hbox {mL min}^{-1}$ (Davies and Morris 1993) and $\mathrm {BW}_\mathrm {ref}= 0.25\,\hbox {kg}$. Since the data set did not comprise very old rats, we did not include any additional age-related effects. For humans, we scaled GFR based on body surface area (BSA) via $\mathrm {GFR}= \mathrm {SF}_\mathrm {unit} \times \mathrm {GFR}_\mathrm {ref}\times ( \mathrm {BSA}/ \mathrm {BSA}_\mathrm {ref})$ with $\mathrm {GFR}_\mathrm {ref}= 105\,\hbox {mL min}^{-1}$ and $\mathrm {BSA}_\mathrm {ref}= 1.73\,\hbox {m}^{2}$ (Poggio et al. 2009). For both cases, the scaling factor $\mathrm {SF}_\mathrm {unit} = 60/1000$ accounts for unit transformation to $\hbox {L h}^{-1}$. For BW and BSA for the reference individuals, see Table 1.

According to the study designs, we chose appropriate reference individuals for scaling. Table 1 lists age, weight, volumes, and densities of the reference individuals. If any of the covariates was not reported in the original study, the missing variables were imputed as described in the supplementary material (Fig. S3).

Urine concentrations were converted to cumulative amounts excreted in urine. This conversion assumed a complete urine collection. When urine collection was incomplete, we omitted urine data time series (see report on dataset curation).

**Table 1:** Physiological parameters of reference individuals

	Human		Rat	
	Male	Female	Young	Old
Body weight in kg; body height in m				
$\mathrm {BW}$	73	60	0.25	0.48
$\mathrm {BH}$	1.76	1.63	–	–
Body surface area in $\hbox {m}^{2}$				
$\mathrm {BSA}$	1.90	1.66	–	–
Haematocrit value				
$\mathrm {Hct}$	0.43	0.38	0.43	0.43
Total vascular blood volume in L				
$V_\mathrm {blo}$	5.30	3.90	0.0160	0.0304
Tissue volumes (interstitial + cellular) in L				
$V_\mathrm {liv}$	1.80	1.40	0.00920	0.0174
$V_\mathrm {spl}$	0.150	0.130	0.000500	0.000950
$V_\mathrm {mus}$	29.0	17.5	0.101	0.192
$V_\mathrm {bon}$	8.07	6.00	0.0140	0.0267
$V_\mathrm {bra}$	1.45	1.30	0.00140	0.00270
$V_\mathrm {kid}$	0.310	0.275	0.00180	0.00350
$V_\mathrm {rob}$	25.8	29.4	0.104	0.200
Tissue densities kg/L				
$$D_\mathrm {liv}= D_\mathrm {spl}= D_\mathrm {kid}= D_\mathrm {mus}= D_\mathrm {bra}= 1$$				
$D_\mathrm {bon}$	1.3	1.3	1.3	1.3
$D_\mathrm {rob}$	0.95	0.94	0.98	0.97
Blood flows L/h				
$Q_\mathrm {liv}$	99.5	95.6	0.867	1.404
$Q_\mathrm {spl}$	11.7	10.62	0.0997	0.161
$Q_\mathrm {mus}$	66.3	42.5	1.39	2.24
$Q_\mathrm {bon}$	19.5	17.7	0.608	0.984
$Q_\mathrm {bra}$	46.8	42.5	0.0997	0.161
$Q_\mathrm {kid}$	74.1	60.2	0.703	1.14
$Q_\mathrm {rob}$	72.2	85.0	1.22	1.98

‘Young’ and ‘Old’ refer to adult rats with body weight of 250 g and 480 g, respectively

Refs. Huisinga et al. (2012), Brown et al. (1997)

### Training and validation data

We used the curated ^26^Al dataset as the training dataset, see Table 2. All data resulted from single po or iv dose administration of Al citrate or chloride, except for a single human, which received a second dose after 2 years [Steinhausen et al. (2004), volunteer 1 equal to volunteer 4]. Since for this individual, only blood/plasma and urine samples are available, we assumed the data after the second dose to be unaffected by the first dose, and treated them as if they were single-dose data due to the fast plasma PK.

For validation, we used the following study data. A published ^27^Al dataset on the kinetics of iv Al citrate in rats (Weisser et al. 2019) was used as a first validation dataset. Rats id = 34–39 and id = 40–45 received iv doses of 0.3 or 0.03 mg ^27^Al/kg BW as aqueous solutions of citrate salt, respectively. Based on these doses, the measured plasma concentrations less the individual pre-dose baselines and plasma volume of $12.9\,\hbox {mL}$ (scaled for $\mathrm {BW}=350\,\hbox {g}$) were converted into the corresponding fid values. We refer to these data as validation ‘^27^Al in rats’.

The second validation dataset comprised retention data in humans after iv administration of Al citrate (Newton and Talbot 2012), which were also partly published in Priest (2004). Fraction of dose retained relates to the amount Al that has not been excreted after administration. In our model, the amount of retained Al is given as $A_\mathrm {ret}= A_\mathrm {gut}+ A_\mathrm {blo}+ \sum _{\mathrm {tis}}{A_\mathrm {tis}}$; note that $A_\mathrm {gut}=0$ in case of iv administration [as in Newton and Talbot (2012)]. The amount of retained Al may be observed either directly by using full-body monitoring devices (Newton and Talbot 2012; Priest 2004) or indirectly via summation of the excreted Al in urine and faeces (Talbot et al. 1995). The dose is typically known. All data were extracted as described in the “Data extraction from references” section. We refer to these data as validation ‘^26^Al full-body retention in humans’.

After the main structure of our model was developed, the results by de Ligt et al. (2018) became available and were rendered suitable as validation data set for human iv plasma predictions by fulfilling the requirements described in the literature search. 14 women received intravenously 2.287 ng of ^26^Al as citrate salt. We determined the corresponding fid values based on the dose and blood volume of 3.6 L, as estimated for female humans with $\mathrm {BW}=60\,\hbox {kg}$. We refer to these fid data as validation ‘^26^Al in humans’.

All datasets and calculations are provided in the Supplementary Material.

### Model structure of the physiology-based toxicokinetic model

**Fig. 1:**
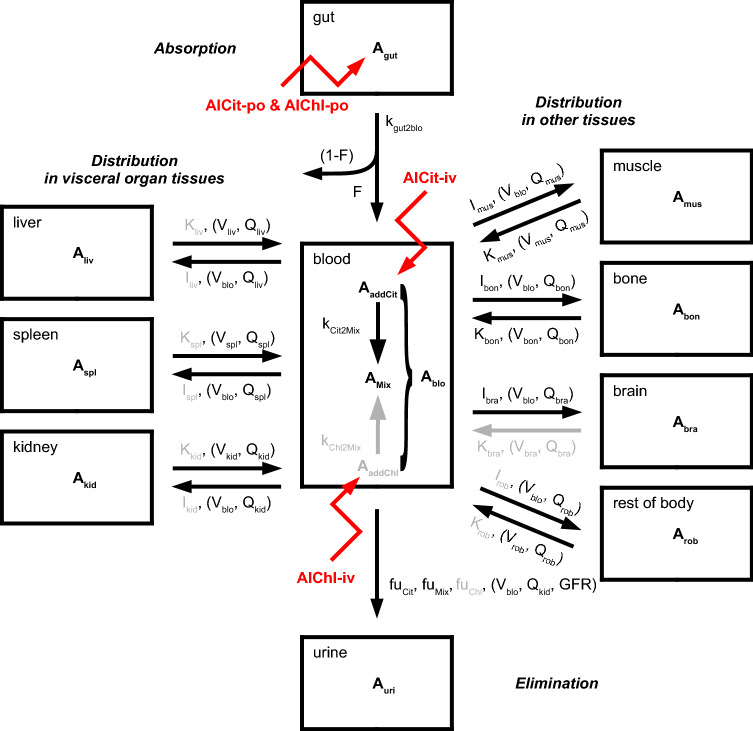
Detailed (black and grey) and simplified (black) PBTK model structure. Amount of Al in body tissues and fluids denoted by *A*. Mass transfer indicated by black arrows with corresponding substance-dependent and -independent (i.e., species-specific) parameters (the latter being stated in brackets). In the blood compartment, different Al species are considered: added, i.e., iv-administered, citrate (addCit), and chloride (addChl) salts as well as a ‘mixed’ state, where all Al species—including transferrin bound Al—are assumed to be in quasi-steady state (Mix). Routes of administration are indicated by red arrows, and comprise intravenous (iv) and per oral (po) administration with bioavailability *F*. Parameters $K_\mathrm {tis}$, $V_\mathrm {tis}$, $Q_\mathrm {tis}$, and $I_\mathrm {tis}$ represent retention coefficients, tissue volumes, organ blood flows, and tissue uptake coefficients, respectively (see Eqs.(5)–(6), Table 3 and text below)

The PBTK model accounts for Al kinetics in plasma ($\mathrm {pla}$), blood ($\mathrm {blo}$), liver ($\mathrm {liv}$), spleen ($\mathrm {spl}$), muscle ($\mathrm {mus}$), bone ($\mathrm {bon}$), brain ($\mathrm {bra}$), kidney ($\mathrm {kid}$), and urine ($\mathrm {uri}$). A ‘rest of body’ compartment ($\mathrm {rob}$) describes the sum of the remaining body spaces like carcass, adipose tissue, and lung, as well as sites escaping quantification in before-mentioned tissue homogenates. The term *tissue* refers to the sum of interstitial and cellular space, thus excluding the vascular space, i.e., peripheral blood. The organs are in exchange via a central blood compartment that includes the arterio-venous space as well as the vascular space of the tissues. An overview of the detailed model structure is given in Fig. 1 (with black and grey parts).

The amount of Al in the different tissues and blood was denoted by $A_\text {cmt}$, where ‘cmt’ denoted the corresponding compartment. Time was denoted by *t*. For associated units, see Table 3.

### Parametrisation of absorption, distribution, and elimination

Oral absorption from the gut was modelled as a first-order process

3 $$\begin{aligned} \frac{\mathrm {d}}{\mathrm {d}t} A_\mathrm {gut}&= -k_\mathrm {{gut}2{blo}} \times A_\mathrm {gut}, \end{aligned}$$

where $k_\mathrm {{gut}2{blo}}$ denotes the absorption rate constant. The total amount $A_\mathrm {blo}$ of Al in blood is the sum of different Al chemical species

4 $$\begin{aligned} A_\mathrm {blo}&= A_\mathrm {addCit}+ A_\mathrm {addChl}+ A_\mathrm {Mix}, \end{aligned}$$

where $A_\mathrm {addCit}$ and $A_\mathrm {addChl}$ represents Al administered as aqueous Al citrate and Al chloride solutions, respectively. The species $A_\mathrm {Mix}$ represents the mixture of all Al comprising chemical species in the bio-phase (in quasi-steady state), including low (e.g., Al citrate) and high molecular weight (e.g., Al transferrin) complexes. We assumed that the administered citrate and chloride species redistribute into the mixture of all Al species with rate constants $k_\mathrm {{\mathrm {Cit}}2{\mathrm {Mix}}}$ and $k_\mathrm {{\mathrm {Chl}}2{\mathrm {Mix}}}$.

We modelled tissue distribution by accounting for Al *uptake* and *release*, without distinguishing between Al species due to lack of sufficient knowledge and data on speciation within tissues. Uptake includes extravasation and cellular uptake, described by an uptake rate constant

5 $$\begin{aligned} k_\mathrm {{\mathrm {blo}}2{\mathrm {tis}}} = \frac{I_\mathrm {tis}\times Q_\mathrm {tis}}{V_\mathrm {blo}}, \end{aligned}$$

while release comprising intravasation and cellular efflux is described by a release rate constant

6 $$\begin{aligned} k_\mathrm {{\mathrm {tis}}2{\mathrm {blo}}}&= \frac{Q_\mathrm {tis}}{ K_\mathrm {tis}\times V_\mathrm {tis}}. \end{aligned}$$

Both rate constants were parametrised in terms of *substance-dependent* and *species-specific* parameters. Substance-dependent parameters were considered unknown and were estimated or fixed. These parameters included the tissue uptake coefficient $I_\mathrm {tis}$ and retention coefficient $K_\mathrm {tis}$ (see Table 3 for parameter units and values). Species-specific parameters were fixed to literature values for reference individuals and included blood volume $V_\mathrm {blo}$, tissue volumes $V_\mathrm {tis}$, and organ blood flows $Q_\mathrm {tis}$ (see Table 1).

We modelled the rate of change of the amount of Al in blood and tissues $\mathrm {tis}\in \{ \mathrm {liv}, \mathrm {spl}, \mathrm {mus}, \mathrm {bon}, \mathrm {bra}, \mathrm {kid}, \mathrm {rob}\}$ by the following system of ordinary differential equations:

7 $$\begin{aligned} \frac{\mathrm {d}}{\mathrm {d}t} A_\mathrm {addCit}&= -k_\mathrm {{\mathrm {Cit}}2{\mathrm {Mix}}} \times A_\mathrm {addCit}- \frac{\mathrm {CL}}{V_\mathrm {blo}} \times A_\mathrm {addCit}\nonumber \\&\quad +\frac{A_\mathrm {addCit}}{A_\mathrm {blo}} \sum _{\mathrm {tis}}{k_\mathrm {{\mathrm {tis}}2{\mathrm {blo}}} \times A_\mathrm {tis}} \nonumber \\&\quad -\sum _{\mathrm {tis}}{k_\mathrm {{\mathrm {blo}}2{\mathrm {tis}}} \times A_\mathrm {addCit}} \end{aligned}$$

8 $$\begin{aligned} \frac{\mathrm {d}}{\mathrm {d}t} A_\mathrm {addChl}&= -k_\mathrm {{\mathrm {Chl}}2{\mathrm {Mix}}} \times A_\mathrm {addChl}- \frac{\mathrm {CL}}{V_\mathrm {blo}}\times A_\mathrm {addChl}\nonumber \\&\quad +\frac{A_\mathrm {addChl}}{A_\mathrm {blo}} \sum _{\mathrm {tis}}{k_\mathrm {{\mathrm {tis}}2{\mathrm {blo}}} \times A_\mathrm {tis}} \nonumber \\&\quad -\sum _{\mathrm {tis}}{k_\mathrm {{\mathrm {blo}}2{\mathrm {tis}}} \times A_\mathrm {addChl}} \end{aligned}$$

9 $$\begin{aligned} \frac{\mathrm {d}}{\mathrm {d}t} A_\mathrm {Mix}&= k_\mathrm {{\mathrm {Cit}}2{\mathrm {Mix}}} \times A_\mathrm {addCit}+k_\mathrm {{\mathrm {Chl}}2{\mathrm {Mix}}} \times A_\mathrm {addChl}\nonumber \\&\quad + F \times k_\mathrm {{gut}2{blo}} \times A_\mathrm {gut}\nonumber \\&\quad +\frac{A_\mathrm {Mix}}{A_\mathrm {blo}} \sum _{\mathrm {tis}}{k_\mathrm {{\mathrm {tis}}2{\mathrm {blo}}} \times A_\mathrm {tis}} \nonumber \\&\quad -\sum _{\mathrm {tis}}{k_\mathrm {{\mathrm {blo}}2{\mathrm {tis}}} \times A_\mathrm {Mix}} - \frac{\mathrm {CL}}{V_\mathrm {blo}} \times A_\mathrm {Mix}\end{aligned}$$

10 $$\begin{aligned} \frac{\mathrm {d}}{\mathrm {d}t} A_{\mathrm {tis}}= k_{{\mathrm {blo}}2{\mathrm {tis}}} \times A_{\mathrm {blo}}-k_{{\mathrm {tis}}2{\mathrm {blo}}} \times A_{\mathrm {tis}}\end{aligned}$$

11 $$\begin{aligned}\frac{\mathrm {d}}{\mathrm {d}t} A_{\mathrm {uri}}= \frac{\mathrm {CL}}{V_{\mathrm {blo}}} \times A_{\mathrm {blo}}, \end{aligned}$$

with bioavailability *F* and clearance $\mathrm {CL}$. Based on Eq. (10), the (steady state) tissue-to-blood fraction of ingested dose ratio was given as

12 $$\begin{aligned} \frac{\mathrm {fid}_\mathrm {tis}}{\mathrm {fid}_\mathrm {blo}} = \frac{A_\mathrm {tis}}{A_\mathrm {blo}} = \frac{k_\mathrm {{\mathrm {blo}}2{\mathrm {tis}}}}{k_\mathrm {{\mathrm {tis}}2{\mathrm {blo}}}} = \frac{I_\mathrm {tis}\times K_\mathrm {tis}\times V_\mathrm {tis}}{V_\mathrm {blo}}. \end{aligned}$$

Since we did not model speciation in the tissues, we assumed that redistribution of Al from the tissue spaces into the blood space did not alter prevailing speciation in blood. Absorbed Al is mainly excreted via urine (Priest 2004), as urinary excretion exceeds faecal excretion by one to two orders of magnitude [e.g., see total amount excreted and excretion rate at 5 and 3250 days post-exposure in Priest (2004)]. Thus, we assumed the faecal route to be negligible and consequently exclusively attributed clearance from the blood to renal elimination into urine. In this process, the effective ultra-filtrable fraction $\mathrm {fu}= \mathrm {fu}(t)$ represents filtration, secretion, and reabsorption processes of Al in the kidneys. As individual Al species differ considerably with respect to filterability (Shirley and Lote 2005), $\mathrm {fu}$ was assumed to depend on the speciation in the blood via

13 $$\begin{aligned} \mathrm {fu}&= \frac{A_\mathrm {addCit}}{A_\mathrm {blo}} \times \mathrm {fu}_\mathrm {Cit}+ \frac{A_\mathrm {addChl}}{A_\mathrm {blo}} \times \mathrm {fu}_\mathrm {Chl}+ \frac{A_\mathrm {Mix}}{A_\mathrm {blo}} \times \mathrm {fu}_\mathrm {Mix}, \end{aligned}$$

with parameters $\mathrm {fu}_\mathrm {Cit}$, $\mathrm {fu}_\mathrm {Chl}$, and $\mathrm {fu}_\mathrm {Mix}$. Renal clearance $\mathrm {CL}=\mathrm {CL}(t)$ of Al from the blood was then defined as

14 $$\begin{aligned} \mathrm {CL}&= \frac{Q_\mathrm {kid}\times \mathrm {fu}\times \mathrm {GFR}}{Q_\mathrm {kid}+ \mathrm {fu}\times \mathrm {GFR}}, \end{aligned}$$

where $\mathrm {GFR}$ is the species-specific glomerular filtration rate. Clearance thus depends on the chemical speciation in blood (through effective ultrafiltrable fraction), kidney blood flow, and $\mathrm {GFR}$. Adding up Eqs. (7)–(9) yielded

15 $$\begin{aligned} \begin{aligned} \frac{\mathrm {d}}{\mathrm {d}t} A_\mathrm {blo}&= k_\mathrm {{gut}2{blo}} \times F \times A_\mathrm {gut}-\mathrm {CL}\times \frac{ A_\mathrm {blo}}{V_\mathrm {blo}} \\&\qquad + \sum _{\mathrm {tis}}{k_\mathrm {{\mathrm {tis}}2{\mathrm {blo}}} \times A_\mathrm {tis}} -\sum _{\mathrm {tis}}{k_\mathrm {{\mathrm {blo}}2{\mathrm {tis}}} \times A_\mathrm {blo}}; \end{aligned} \end{aligned}$$

therefore, $\mathrm {CL}$ represents the average clearance applying to all Al species. The amount of Al in plasma was derived from $A_\mathrm {blo}$ according to

16 $$\begin{aligned} A_\mathrm {pla}= \frac{A_\mathrm {blo}\times (1-\mathrm {Hct})}{\mathrm {BP}} \end{aligned}$$

with haematocrit value $\mathrm {Hct}$ and the blood-to-plasma ratio $\mathrm {BP}$.

### Parameter estimation and implementation

We used the framework of non-linear mixed-effect modelling to account for inter-study and inter-individual variability (e.g., Lavielle and Bleakley 2015). In this approach, the structural parameters (i.e., those linked to the PBTK model structure) are assumed to vary between different studies (for rats) or individuals (for humans), comprising a fixed effect (population mean) and a random effect (variation between studies/individuals). For intrinsically positive structural parameters, we assumed a log-normal distribution and for fraction parameters (between 0 and 1), and we assumed a logit-normal distribution. The (hyper-)parameters specifying these distributions were estimated. Precision of parameter estimates was quantified as relative standard error (R.S.E.). Shrinkage of the random effects was determined based on the empirical and estimated variance of the random effects, as described in Lavielle and Bleakley (2015). A low shrinkage value is required for unbiased empirical Bayes estimates (EBEs).

To obtain approximately homoscedastic distributions of the residuals and to avoid negative predictions, we log-transformed both the observations and the model predictions (Mould and Upton 2013). We used an additive error model on the transformed scale, which corresponds to a log-normal multiplicative error model on the original scale.

We tested and compared two different approaches to choose physiological parameters (blood flows and organ volumes) for a study/an individual: (i) an allometric scaling approach using body weight for rats and body weight/body height for humans (Huisinga et al. 2012); and (ii) choosing physiological parameters from appropriate reference individuals from Table 1, where for each individual/animal, the closest reference individual out of the set of four—human adults (male/female) and rats (young/old based on body weight) was chosen.

For data handling and plotting, we used Matlab 2015a (8.5.0.197613) by MathWorks. Parameter estimation was performed in Monolix 2016R1. To improve convergence of Monolix estimation in the presence of a large number of parameters and observables, we increased the default number of Markov chains from 1 to 25 and used the stiff flag to solve the system of differential equations. After estimation in Monolix, simulations with the estimated population parameters were run with R version 3.4.1 and package mlxR version 3.2.0. We assessed sensitivity of parameter estimates (i) with respect to starting values, by repeating the estimation procedure five times from over-dispersed random initial estimates, and (ii) with respect to the de-aggregation step (see “Methods”), by repeating the estimation procedure for four additional de-aggregated datasets, generated in an independent way. Further details about the development environment, all scripts, project files, and raw data are provided as supplementary material.

For a graphical representation of model predictions, we performed 250 Monte Carlo simulations using the estimated population parameters and inter-individual variability, depicting the simulation results as percentile plots. In addition, training data and individual predictions based on the EBEs are shown for each individual identifier. For a better overview, we stratified training dataset and model predictions based on species and administered Al salt. For each stratified plot, two prediction intervals are shown, one for iv and one for po dosing.

## Results

### Identification of a comprehensive dataset for PBTK model development

We used the identified ^26^Al studies reported in the 12 (human) and 30 (animal) references in Weisser et al. (2017, Tables 1 and 2) as a starting point for the identification of a comprehensive dataset. The two most recent studies in de Ligt et al. (2018) and in Jugdaohsingh et al. (2000) were considered as potential independent validation data for model development.

With respect to animal species, rats were used in the vast majority of studies, while only two studies (Flarend et al. 1997; Radunović et al. 1997) used mice and rabbits. The two studies were subsequently disregarded. Regarding the routes of administration and formulations, iv and/or po administration of aqueous solutions of chloride (AlChl) and citrate (AlCit) salts were most abundant (11/12 human and 19/30 animal references). The remaining references report about dermal (Flarend et al. 2001), subcutaneous (Amevor et al. 1994; Amevor 1995; Yumoto et al. 2000, 2001, 2003, 2004) or intra-peritoneal studies (Yumoto et al. 1997), or used unspecified/non citrate or chloride salts (Yokel and Florence 2006, 2008; Yokel et al. 2008; Fink et al. 1994). From a PBTK model development point of view, dermal, intra-peritoneal, and subcutaneous administrations are challenging due to the complex and partly unknown absorption process. These studies were subsequently excluded.

We further focussed on healthy individuals and excluded all studies with Alzheimer and glomerular nephritis patients due to a limited quantitative understanding of the impact of such disease effects. Some studies report about special treatment like iron-deficient diet, etc. Due to lack of knowledge on the impact of most special treatments, we disregarded all but the following special treatments: co-administration of additional citrate, water (hardwater or softwater), and/or restrainment from food (fasted).

We generally aimed for the original references, i.e., references that first report about a study. We deviated from the rule, however, in case of alternative references that included higher quality figures, reported in more detail about the study design, or did not show inconsistencies that were present in the original publication. This leads to the inclusion of the diploma thesis by Beck (1997) and the articles by Kobayashi et al. (1990), Steinhausen et al. (1996), Nolte et al. (2001). Finally, we decided to exclude the studies on whole-body retention (Priest 2010; Newton and Talbot 2012; Talbot et al. 1995), since these are less suited for PBTK model development, but may better be used for model validation.

### Curation of the comprehensive dataset

After identification of the comprehensive dataset, we performed an in-depth quality control of the corresponding data. In some cases, this led to the exclusion of further samples or subjects. As a result, we obtained a comprehensive curated dataset of human and rat ^26^Al studies (see Table 2).

**Fig. 2:**
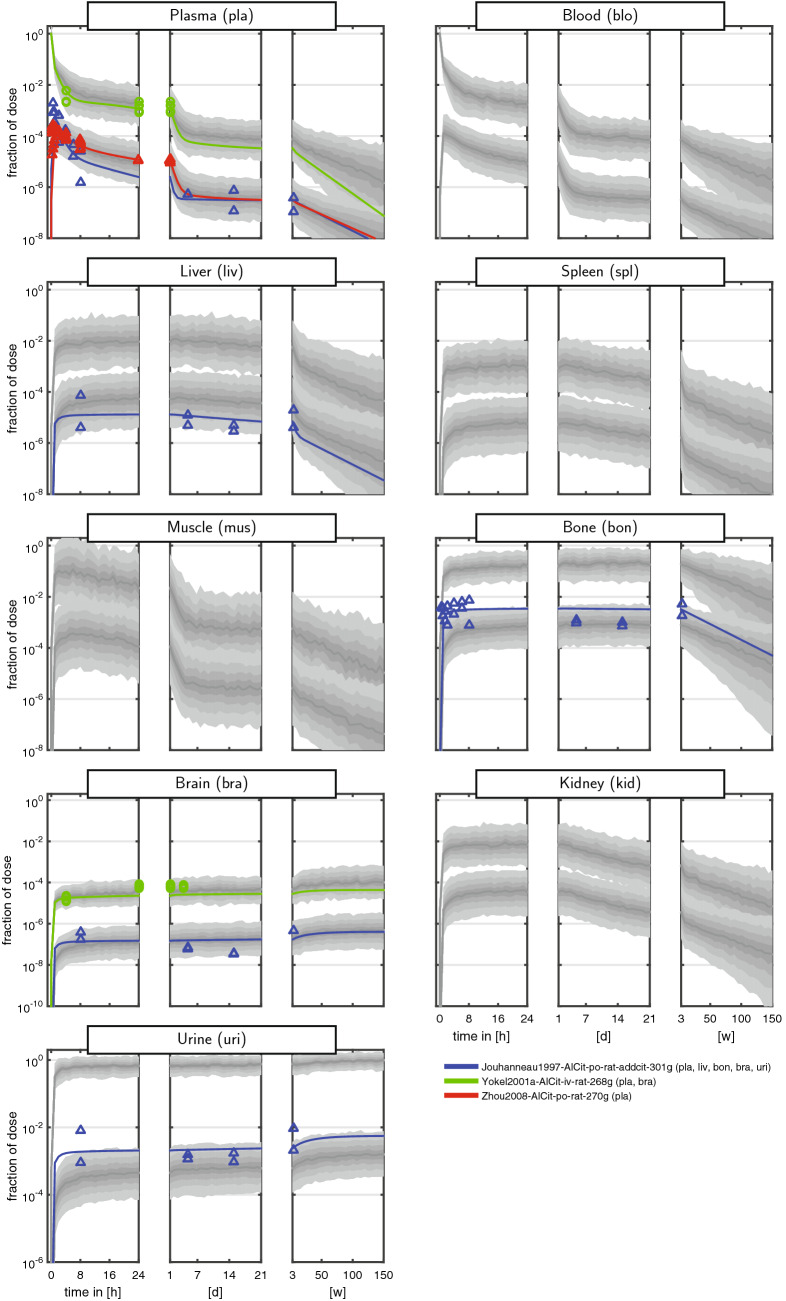
Aluminium disposition in *rats* after intravenous (circles) and oral (triangles) single-dose administration of aqueous solutions of *Al citrate*. Colours link to the legend, where the identifier and measured tissues and body fluids are summarised. The shaded areas are the median and the central 20th, 40th, 60th and 80th percentiles of the population predictions (based on 250 Monte Carlo simulations). Coloured solid lines are individual predictions based on the empirical Bayes estimates. Upper and lower band refer to iv and po administration, respectively (colour figure online)

**Fig. 3:**
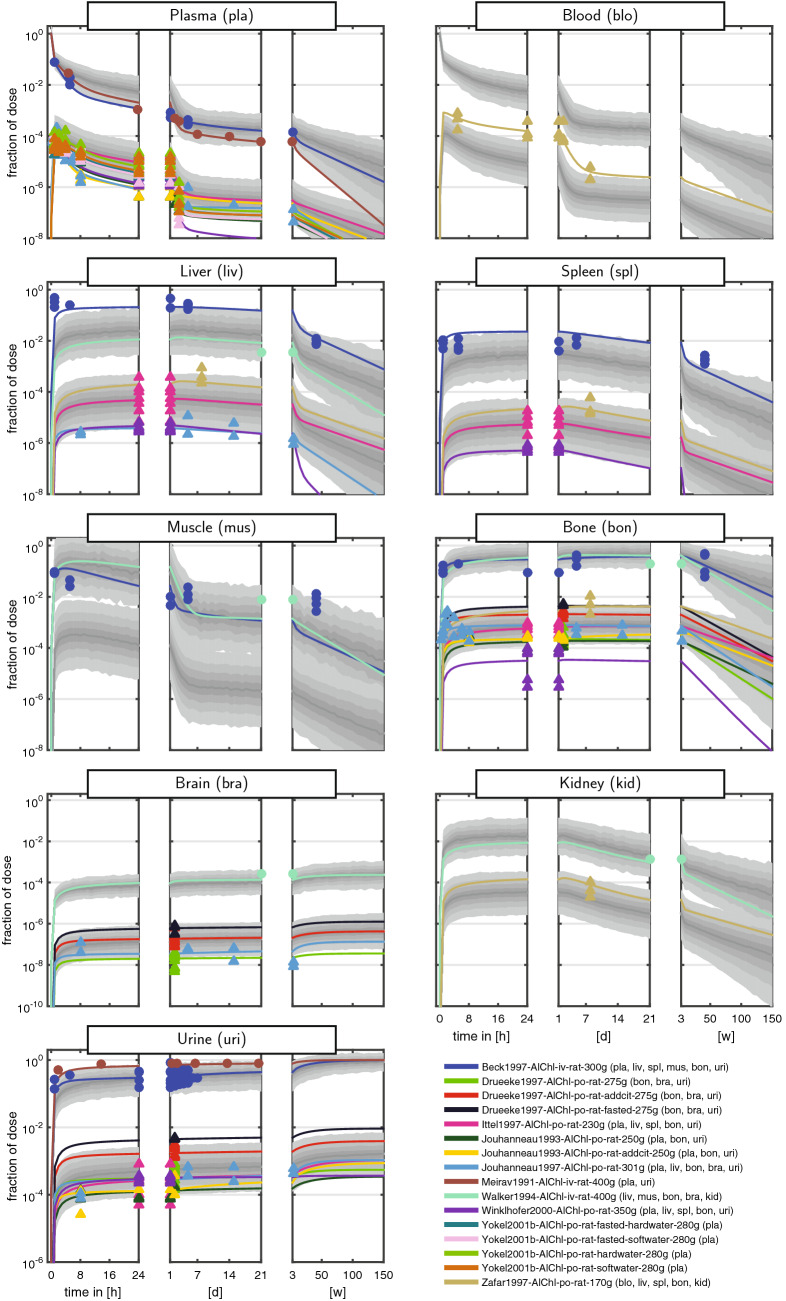
Aluminium disposition in *rats* after intravenous (circles) and oral (triangles) single-dose administration of aqueous solutions of *Al chloride*. Colours link to the legend, where the identifier and measured tissues and body fluids are summarised. The shaded areas are the median and the central 20th, 40th, 60th and 80th percentiles of the population predictions (based on 250 Monte Carlo simulations). Coloured solid lines are individual predictions based on the empirical Bayes estimates. Upper and lower band refer to iv and po administration, respectively (colour figure online)

In the study by Zafar et al. (1997), we found contradicting values for the time-points of the measurements in figures and text. By interpreting the data in the light of all other data (see Figs. 2 and 3), we assumed that the values from the text are correct. In the same study, in Fig. 1 and 2, units are given in % dose per g tissue. However, as scaling resulted in $>400 \%$ dose recovery in bone, we assumed that the authors plotted % dose per kg tissue instead. This assumption was supported by the fact that liver, spleen, and kidney samples of this study were then within the range of comparable studies, as shown in Figs. 2 and 3.

The rat data published in Beck (1997) were later re-published together with human data in Steinhausen (1997), Steinhausen et al. (2004). We included the original rat data by Beck in the dataset, as they covered additional 0–24 h cumulative urine samples. However, due to incomplete urine collection for individual rats, some of the urine concentrations from Beck (1997) could not be converted to cumulative amount excreted in urine and were thus excluded from the dataset. Since this affected only 6 urine samples between 0 and 120 h, we considered the impact on data analysis as low. Information on dosing was complemented from Steinhausen et al. (2004). The dissertation by Steinhausen (1997) comprised some inconsistencies, such as a mix-up between the 79 and 84 kg volunteer. For the human data, we therefore included the re-published data from Steinhausen et al. (2004).

Although not explicitly mentioned, the studies reported in Meirav et al. (1990, 1991) were identified as identical. We maintained the more recent reference in the dataset. Moreover, we identified the studies Meirav et al. (1991) and Walker and Sutton (1994) to refer to the same study. While the first published the plasma and urine samples, the second reported the tissue samples. The ^27^Al dose was complemented from Walker and Sutton (1994). As both datasets were presumingly independently analysed and had no overlapping observations, we maintained both and allocated separate identifiers for both studies.

All rats in the study by Zhou et al. (2008) were additionally dosed with an iv ^27^Al infusion (100 $\mu$g Al per kg BW per h) from 14 h prior and 24 h after the ^26^Al dosing. As additional ^27^Al exposure was not controlled in the other studies, we did not expect these infusions to bias the results with respect to the tracer kinetics and did not exclude the study from the dataset. Along the same line of argument, we did not exclude Drueeke et al. (1997), King et al. (1997) despite low and medium silicate exposure (i.e., in these cases, silicate exposure was not considered as special treatment). Yokel et al. (2001b) explicitly used fasted animals and tested water with different mineral content. These conditions were not controlled in other studies; we rather considered this variation as an enriching element to the overall variability.

The majority of observations were reported in the unit ‘fraction of ingested dose’ (fid). The second most abounded unit reported was g/L or some derived unit, like pg/L, fg/ml). Quantification of the Al amount deposited in the individual tissues and body fluids is based on ^26^Al quantification in sample matrices from rats and humans. Samples of organs like liver, spleen, brain, and kidney typically originate from homogenised aliquots of larger parts of the organ or the organ as a whole. Samples from muscle tissue originate from a specific part, namely the thigh, consistently over all studies. For bone, measurements generally result from femur samples (Beck 1997; Drueeke et al. 1997; Jouhanneau et al. 1993, 1997; Walker and Sutton 1994; Winklhofer et al. 2000; Zafar et al. 1997), except for Ittel et al. (1997), where samples from tibia bone are analysed. Beyond the origin of the bone sample, also sample preparation varied between studies: in two studies, the bone marrow was removed (Beck 1997; Winklhofer et al. 2000), while all other studies did not mention such procedure. The conversion of units to fid was successfully for all references except Moore et al. (2000), where we were not able to convert the unit ‘relative uptake factor’ to fid. It was thus excluded.

**Table 2:** Comprehensive curated dataset of ^26^Al kinetic data. For composition of identifier and abbreviations, see text

Identifier	Number of samples in tissues									Dose in	Duration	Reported unit	
	pla	blo	liv	spl	mus	bon	bra	kid	uri	mol/kg	in days	Type	References
Edwardson1993-AlCit-po-hum-73kg	10	–	–	–	–	–	–	–	–	1E–5	0 (5 h)	g/L	Edwardson et al. (1993)
Fifield1997-AlCit-po-hum-73kg	10	–	–	–	–	–	–	–	34	4E–7	25	fid	Fifield (1997)
King1997-AlCit-po-hum-73kg	22	–	–	–	–	–	–	–	23	4E–6	5	fid	King et al. (1997)
Moore1997-AlCit-po-hum-60kg	2	–	–	–	–	–	–	–	–	6E–8	0 (1 h)	g/L	Moore et al. (1997)
Moore1997-AlCit-po-hum-73kg	2	–	–	–	–	–	–	–	–	5E–8	0 (1 h)	g/L	Moore et al. (1997)
Priest1995-AlCit-iv-hum-77kg	–	17	–	–	–	–	–	–	–	2E–7	891	fid/g	Priest et al. (1995)
Priest1996-AlCit-po-hum-73kg	–	6	–	–	–	–	–	–	–	4E–2	1	g/L	Priest et al. (1996)
Priest1998-AlChl-po-hum-62kg	–	3	–	–	–	–	–	–	–	6E–6	1	g/L	Priest et al. (1998)
Priest1998-AlChl-po-hum-68kg	–	3	–	–	–	–	–	–	–	5E–6	1	g/L	Priest et al. (1998)
Steinhausen2004-AlChl-iv-hum-79kg	18	–	–	–	–	–	–	–	12	8E–7	163	fid	Steinhausen et al. (2004)
Steinhausen2004-AlChl-iv-hum-80kg	19	–	–	–	–	–	–	–	12	8E–7	328	fid	Steinhausen et al. (2004)
Steinhausen2004-AlChl-po-hum-75kg	20	–	–	–	–	–	–	–	12	4E–6	65	fid	Steinhausen et al. (2004)
Steinhausen2004-AlChl-po-hum-79kg	15	–	–	–	–	–	–	–	4	4E–6	11	fid	Steinhausen et al. (2004)
Steinhausen2004-AlChl-po-hum-84kg	15	–	–	–	–	–	–	–	7	4E–6	23	fid	Steinhausen et al. (2004)
Talbot1995-AlCit-iv-hum-65kg	–	4	–	–	–	–	–	–	5	3E–8	5	fid/L & fid	Talbot et al. (1995)
Talbot1995-AlCit-iv-hum-73kg	–	4	–	–	–	–	–	–	5	3E–8	6	fid/L & fid	Talbot et al. (1995)
Talbot1995-AlCit-iv-hum-74kg	–	4	–	–	–	–	–	–	5	3E–8	5	fid/L & fid	Talbot et al. (1995)
Talbot1995-AlCit-iv-hum-75kg	–	4	–	–	–	–	–	–	5	3E–8	5	fid/L & fid	Talbot et al. (1995)
Talbot1995-AlCit-iv-hum-76kg	–	3	–	–	–	–	–	–	5	3E–8	5	fid/L & fid	Talbot et al. (1995)
Talbot1995-AlCit-iv-hum-90kg	–	4	–	–	–	–	–	–	5	2E–8	5	fid/L & fid	Talbot et al. (1995)
Beck1997-AlChl-iv-rat-300g	10	–	13	14	14	12	–	–	27	2E–6	292	fid	Beck (1997)
Drueeke1997-AlChl-po-rat-275g*	–	–	–	–	–	16	8	–	16	7E–6	2	fid	Drueeke et al. (1997)
Drueeke1997-AlChl-po-rat-addcit-275g*	–	–	–	–	–	8	8	–	8	7E–6	2	fid	Drueeke et al. (1997)
Drueeke1997-AlChl-po-rat-fasted-275g*	–	–	–	–	–	8	4	–	8	7E–6	2	fid	Drueeke et al. (1997)
Ittel1997-AlChl-po-rat-230g*	6	–	7	6	–	6	–	–	6	2E–2	1	g/L & g/g	Ittel et al. (1997)
Jouhanneau1993-AlChl-po-rat-250g	6	–	–	–	–	6	–	–	6	7E–6	2	fid	Jouhanneau et al. (1993)
Jouhanneau1993-AlChl-po-rat-addcit-250g	6	–	-	–	–	6	–	–	6	7E–6	2	fid	Jouhanneau et al. (1993)
Jouhanneau1997-AlChl-po-rat-301g	19	–	8	–	–	20	8	–	8	6E–6	30	fid	Jouhanneau et al. (1997)
Jouhanneau1997-AlCit-po-rat-addcit-301g	20	–	8	–	–	20	7	–	8	6E–6	30	fid	Jouhanneau et al. (1997)
Meirav1991-AlChl-iv-rat-400g	7	–	–	–	–	–	–	–	7	3E–6	21	fid	Meirav et al. (1991)
Walker1994-AlChl-iv-rat-400g	–	–	1	–	1	1	1	1	–	3E–6	21	fid/g	Walker and Sutton (1994)
Winklhofer2000-AlChl-po-rat-350g*	5	-	5	5	-	5	-	–	5	2E–2	1	fid	Winklhofer et al. (2000)
Yokel2001a-AlCit-iv-rat-268g*	10	–	–	–	–	–	15	–	–	1E–6	4	fid/g	Yokel et al. (2001a)
Yokel2001b-AlChl-po-rat-fasted-hardwater-280g*	30	–	–	–	–	–	–	–	–	7E–5	3	g/L	Yokel et al. (2001b)
Yokel2001b-AlChl-po-rat-fasted-softwater-280g*	30	–	–	–	–	–	–	–	–	7E–5	3	g/L	Yokel et al. (2001b)
Yokel2001b-AlChl-po-rat-hardwater-280g*	30	–	–	–	–	–	–	–	–	7E–5	3	g/L	Yokel et al. (2001b)
Yokel2001b-AlChl-po-rat-softwater-280g*	24	–	–	–	–	–	-	–	–	7E–5	3	g/L	Yokel et al. (2001b)
Zafar1997-AlChl-po-rat-170g*	–	12	3	3	–	3	–	3	–	4E–1	8	fid/g	Zafar et al. (1997)
Zhou2008-AlCit-po-rat-270g	46	–	–	–	–	–	–	–	–	2E–4	1	fid/L	Zhou et al. (2008)
Sums of samples and dose ranges	382	64	45	28	15	111	51	4	239	2E–8 to 3E–6 and 5E–8 to 4E–1 mol/kg for iv and po			

The dose refers to the sum of administered ^26^Al and ^27^Al amounts. Identifiers with * label datasets reported as summary statistics, which were de-aggregated

A total of seven studies reported summary data instead of individual observations (Drueeke et al. 1997; Ittel et al. 1997; Schoenholzer et al. 1997; Winklhofer et al. 2000; Yokel et al. 2001a, b; Zafar et al. 1997). Since Schoenholzer et al. (1997) reported no information on the variance of the aggregated data (mean of 9 rats), we excluded this study, since the variance information is crucial for data de-aggregation. All remaining six studies (all in rat) reported mean and standard deviation. De-aggregation of summary data (identifiers marked with * in Table 2) affected roughly a quarter of all observations (272/939). When estimating parameters during model development, the impact of this randomness on these estimates should be explicitly addressed.

The final comprehensive curated dataset (in short, curated dataset) contained 319 human and 620 rat ^26^Al observations. A summary of the dataset is given in Table 2; the dataset is provided as supplementary material. Additionally, we provide an interactive Shiny application (Chang 2020) that allows to explore the datasets visually by subsetting, stratifying, or grouping according to arbitrary dataset columns.

### Non-curated digitized dataset

During the data compilation process, we digitized and extracted many more data from the reported references than we finally included in the curated dataset. For example, non-healthy individuals were typically extracted in one go along with the healthy control individuals from the same figure. We decided to make all data that we extracted available to the community in the so-called *non-curated digitized dataset* for further exploration. Data finally included in the curated dataset are correspondingly flagged (in addition, we also provide a separate file with the curated dataset only).

We do not take any responsibility of correctness and completeness of the data presented in the non-curated digitized dataset. Thus, before using this dataset, steps of quality control should be taken. All reported references/studies of non-curated but digitized data that are not part of the curated dataset defined in Table 2 are listed in the supplementary material (Tables S1–S3).

### Characteristics of the curated dataset

The curated dataset comprised measurements of the fraction of ingested dose over more than eight orders of magnitude and a time span up to 150 weeks (w). To cope with the huge range of both the dependent and independent variable, data were often shown on a log–log scale (e.g., Steinhausen et al. 2004). These plots, however, are very difficult to interpret, since even a simple multi-exponential decay is very hard to recognise. We therefore introduced a new graphical representation comprising triplets of semi-logarithmic plots covering the first 24 h (left panel), the following days up to the end of the first week (middle panel), and the following weeks up to almost 3 years (right panel). The Al measurements of the curated dataset for rats and humans are shown in Figs. 2 + 3 and 4 + 5, respectively. Additionally, a more compact representation of these data without model predictions is given in the supplement (Figs. S1 + S2).

Overall, the kinetics in tissues and body fluids show a clear separation between iv and po data. As a result of the unit fid, iv data are roughly three orders of magnitude larger compared to po data (reflecting the low oral bioavailability of Al salts). Furthermore, the po data exhibited a tendency to a larger intra- and inter-individual variability compared to iv.

For plasma and blood kinetics, we discerned several consecutive phases (most clearly seen in humans): (i) a fast steep decline of the amount of Al in blood and plasma during the first 4 h post-dose. The half-life of this kinetics is on the time-scale of minutes; (ii) a slower phase from 4 h to 7 days with a largely decreased slope and a half-life on the time-scale of days; (iii) a further slowed down kinetics from 7 days to 50 weeks. In humans, we further observed an even slower phase beyond 50 weeks due to the very long observation time span up to 150 weeks.

Regarding the maximal fid values, we inferred the following ranking: high (bone and liver), medium (muscle and spleen), and low (brain). While bone seems to be consistently high, liver starts to decay on the right panel. Within the rat studies, a large variability is observed. For example, the fid data in liver (and slightly less in spleen) span two orders of magnitude after 24 h, see Fig. 3 [pink triangles corresponding to Ittel et al. (1997)]. This is a result of intra-individual and inter-individual variability, in addition to potential differences in BW, dose, administration, food availability, housing, and scaling of data.

Importantly, the curated dataset allows to set different studies into context. For example, the rat plasma data from Ittel et al. (1997) and Jouhanneau et al. (1997) following po administration of Al chloride show quite different behaviour. The latter data peak higher and decay faster during the first 24 h (left panel), while they stay almost constant over the following days (middle panel). The former data peak lower and subsequently decay more slowly. Each study on its own would result in a distorted picture of the plasma kinetics, and only the totality of data allows to appreciate the large variability after po administration.

### Simplifying assumptions to improve parameter identifiability

Attempting to estimate all parameters of the detailed ten-compartment PBTK model depicted in Fig. 1 (with black and grey parts) solely based on the ^26^Al training dataset in rats and humans resulted in parameter identifiability problems. Therefore, we simplified some model details or fixed some parameter values based on additional prior knowledge of peculiarities of Al TK, as described in the following. The resulting simplified 10-compartment PBTK model is depicted in Fig. 1 (without the parts that are greyed out).

We assumed the amount of Al in erythrocytes to be negligible compared to the amount in plasma, implying $A_\mathrm {pla}= A_\mathrm {blo}$. For the blood-to-plasma ratio, this implies $\mathrm {BP}= (1-\mathrm {Hct})$, with the haematocrit given in Table 1. This assumption seems reasonable in light of reported values of 86% and 90% of Al attributed to the plasma fraction of blood in Priest et al. (1995) and Priest (2004), respectively.

Available experimental data in brain (see Figs. 2 + 3) show no sign of release from the tissue. Therefore, and in line with Priest (2004), this organ was conservatively (from a risk assessment point of view) modelled as a sink, i.e., $k_\mathrm {{bra}2{blo}}=0$ (and formally, $K_\mathrm {bra}= \inf$). In view of the comparable kinetics in liver and spleen (profiles seem to be just shifted), and based on expectations and lack of more detailed knowledge and data, we assumed that the visceral organs liver, spleen, and kidney exhibit comparable uptake and release characteristics. More precisely, the tissue uptake and retention coefficients (see Eqs. (5) and (6)) were assumed to be identical: $I_\mathrm {liv}= I_\mathrm {spl}= I_\mathrm {kid}= I_\mathrm {lsk}$ and $K_\mathrm {liv}= K_\mathrm {spl}= K_\mathrm {kid}= K_\mathrm {lsk}$, using the abbreviation $\mathrm {lsk}~\hat{=}~{\underline{\text {l}}\text {iv}},{\underline{\text {s}}\text {pl}},{\underline{\text {k}}\text {id}}$. Notably, this does not imply identical levels in these organs, since the tissue uptake and retention coefficients are only one factor in the tissue uptake and release rate (see Eqs. (5) and (6)). Due to lack of data for the rest of body compartment, we assumed $I_\mathrm {rob}=K_\mathrm {rob}= 1$.

The detailed model structure with three Al states ($A_\mathrm {addCit}$, $A_\mathrm {addChl}$ and $A_\mathrm {Mix}$) in blood was designed to account for potential differences in renal elimination depending on the route and Al salt (citrate or chloride) administered. The given data, however, did not allow to identify the speciation dynamics in all details. We made the following simplifying assumptions: In contrast to other Al species, Al citrate is known to be fully ultrafiltrable (Shirley and Lote 2005). In addition, the effective ultrafiltrable fraction of Al species in quasi-steady state has been reported to be $\approx 10 \%$ (Steinhausen et al. 2004; Shirley and Lote 2005). To improve parameter identifiability, we used this prior knowledge to fix the ultrafiltrability parameters, $\mathrm {fu}_\mathrm {Cit}=1$ and $\mathrm {fu}_\mathrm {Mix}= 0.1$. Moreover, we assumed the transition from $A_\mathrm {addChl}$ to $A_\mathrm {Mix}$ to be instantaneous (compared to the slower transition from $A_\mathrm {addCit}$ to $A_\mathrm {Mix}$).

This rendered the species $A_\mathrm {addChl}$ and the parameters $\mathrm {fu}_\mathrm {Chl}$ and $k_\mathrm {{\mathrm {Chl}}2{\mathrm {Mix}}}$ obsolete. As a consequence, iv administration of any solutions of chloride salts was directly performed into the $A_\mathrm {Mix}$ compartment.

Based on the curated dataset, we could not inform a salt-specific absorption. Possibly, the large inter-study variability prevented identification of these effects. Thus, in our PBTK model, there is only a single $k_\mathrm {{gut}2{blo}}$ for both salts.

### Estimated model parameters and goodness-of-fit plots

After using the simplifying assumptions stated above, all parameters of the simplified ten-compartment PBTK model depicted in Fig. 1 (without the parts that are greyed out) were identifiable. Parameter estimation from over-dispersed initial estimates, and additionally generated de-aggregated datasets yielded practically identical parameter estimates. Estimated model parameters are listed in Table 3. For most parameters, the estimation was very precise (R.S.E.$\le$ 30%), and only the location parameters for $k_\mathrm {{gut}2{blo}}$, $I_\mathrm {lsk}$, and $k_\mathrm {{Cit}2{Mix}}$ and the variability parameter for $K_\mathrm {bon}$ were slightly less precise (30% < R.S.E.$\le$ 60%). As shrinkage on all parameters was high ($35-91\%$), we focussed the further analyses with the calibrated model on the population predictions, which are unaffected by high shrinkage (Lavielle and Bleakley 2015; Mould and Upton 2013).

The comparison of the use of allometrically scaled physiological parameters vs. parameters of a similar reference individual (see “Methods” section) showed that the latter approach performed comparably well (in terms of predictive performance), while being computationally faster. Therefore, we decided to determine blood flows and organ volumes using the latter approach.

**Fig. 4:**
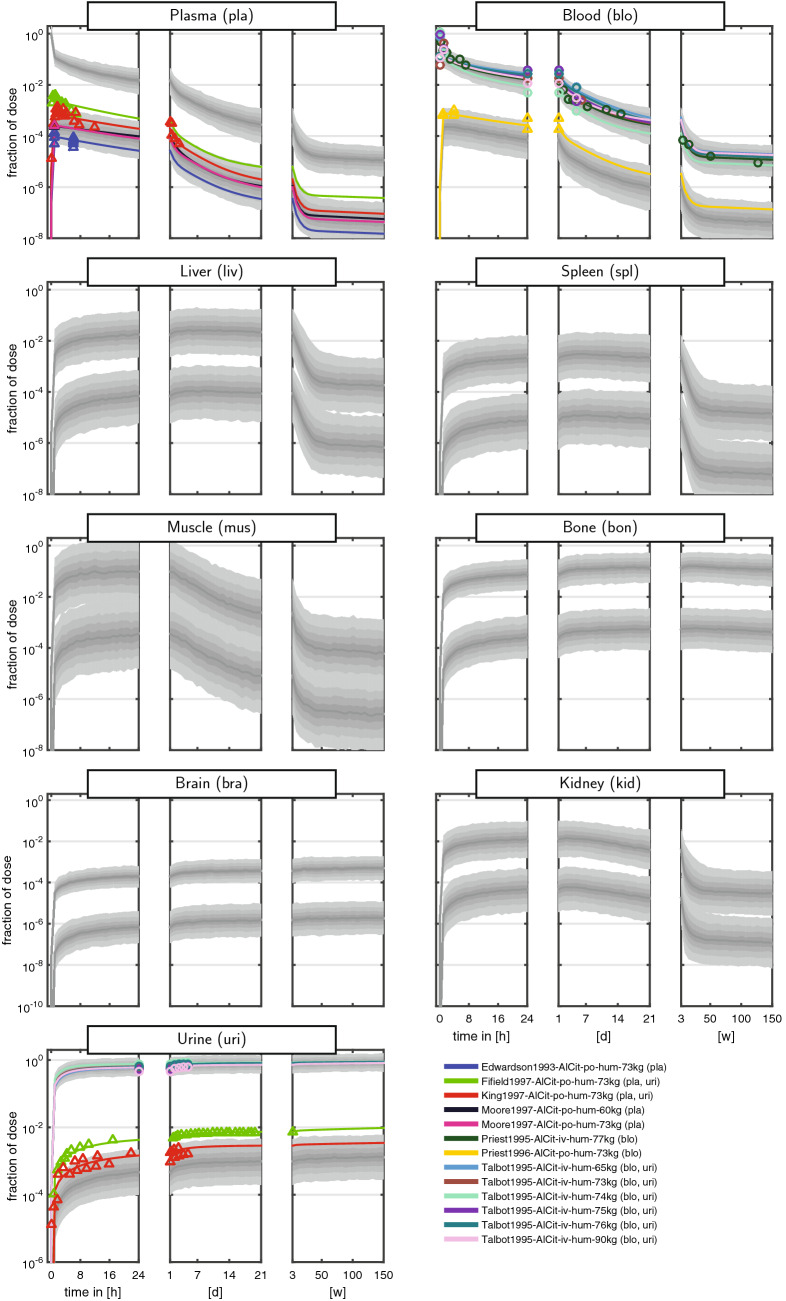
Aluminium disposition in *humans* after intravenous (circles) and oral (triangles) single-dose administration of aqueous solutions of *Al citrate*. Colours link to the legend, where the identifier and measured tissues and body fluids are summarised. The shaded areas are the median and the central 20th, 40th, 60th and 80th percentiles of the population predictions (based on 250 Monte Carlo simulations). Coloured solid lines are individual predictions based on the empirical Bayes estimates. Upper and lower band refer to iv and po administration, respectively (colour figure online)

**Fig. 5:**
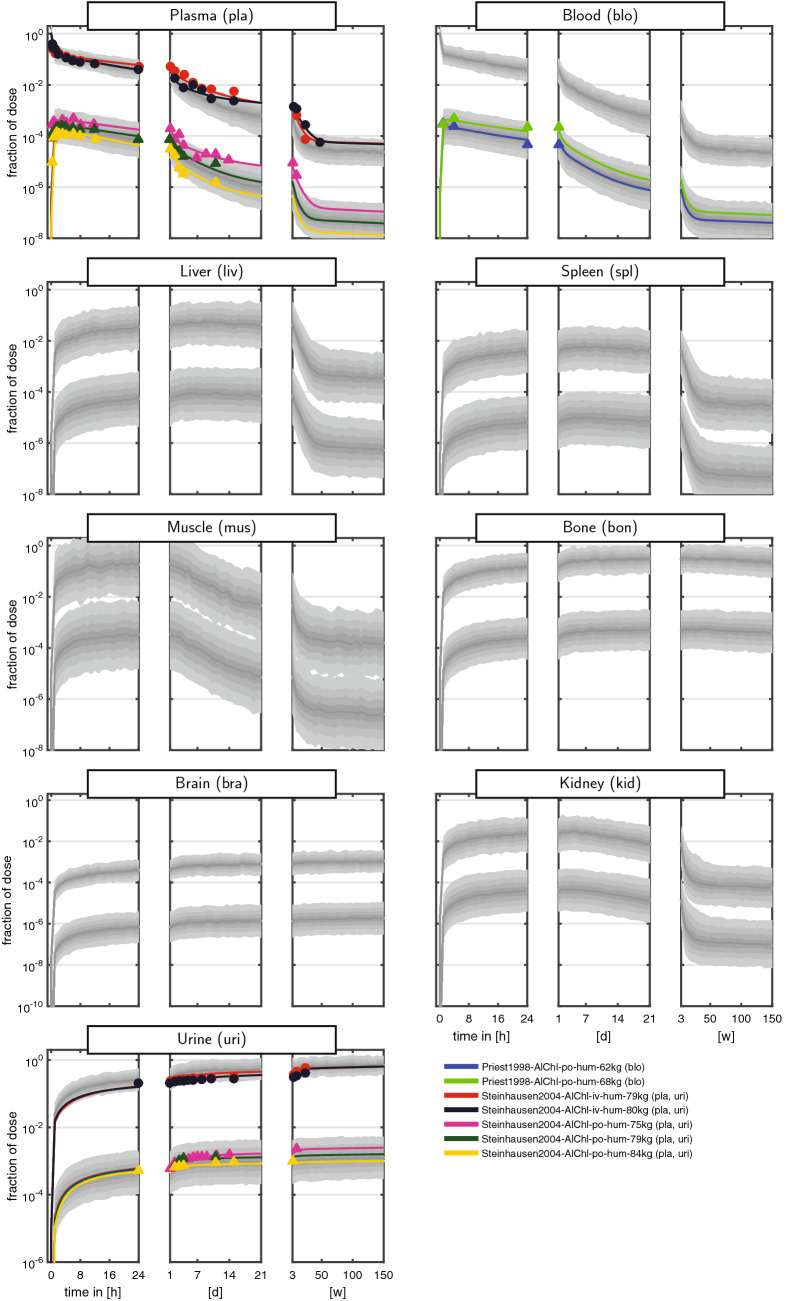
Aluminium disposition in *humans* after intravenous (circles) and oral (triangles) single-dose administration of aqueous solutions of *Al chloride*. Colours link to the legend, where the identifier and measured tissues and body fluids are summarised. The shaded areas are the median and the central 20th, 40th, 60th and 80th percentiles of the population predictions (based on 250 Monte Carlo simulations). Coloured solid lines are individual predictions based on the empirical Bayes estimates. Upper and lower band refer to iv and po administration, respectively (colour figure online)

A graphical representation of the model fits is shown in Figs. 2, 3, 4, and 5. Overall, the data are well represented by the population predictions (median and central 80% percentile range). Note that while for individual studies, an improvement seemed to be possible, this would worsen the fits of other study data. The advantage of the non-linear mixed-effects modelling approach is that all data can be integrated at the same time, balancing the fit of individual study data and the ability to represent all data with a single model.

**Table 3:** Fixed parameter values and parameter estimates, complementing the fixed species-specific parameters of the reference individuals tabulated in Table 1

Parameter	Value	R.S.E. in %	$\omega$	R.S.E. in %
Oral absorption rate constant, $\log (\mathcal {N})$, in $\hbox {h}^{-1}$				
$k_\mathrm {{gut}2{blo}}$	2.43	56	1.34	30
Oral bioavailability, $\mathrm {logit}(\mathcal {N})$, dimensionless				
*F*	0.00182	22	1.03	15
Uptake coefficients, $\mathrm {logit}(\mathcal {N})$, dimensionless				
$I_\mathrm {lsk}$	0.000885	41	1.39	23
$I_\mathrm {mus}$	0.00981	20	0	Fixed
$I_\mathrm {bon}$	0.019	20	0.763	19
$I_\mathrm {bra}$	$2.14\times 10^{-5}$	17	0	Fixed
$I_\mathrm {rob}$	1	Fixed	0	Fixed
Retention coefficients, $\log (\mathcal {N})$, dimensionless				
$K_\mathrm {lsk}$	$5.29\times 10^4$	22	0	Fixed
$K_\mathrm {mus}$	116	16	0	Fixed
$K_\mathrm {bon}$	$1.16\times 10^5$	29	0.556	52
$K_\mathrm {bra}$	$\inf$	Fixed	0	Fixed
$K_\mathrm {rob}$	1	Fixed	0	Fixed
Equilibration rate constant, $\log (\mathcal {N})$, in $\hbox {h}^{-1}$				
$k_\mathrm {{Cit}2{Mix}}$	0.19	32	0	Fixed
Effective ultrafiltrable fractions, $\log (\mathcal {N})$, dimensionless				
$\mathrm {fu}_\mathrm {Cit}$	1	Fixed	0	Fixed
$\mathrm {fu}_\mathrm {Mix}$	0.1	Fixed	0	Fixed
Glomerular filtration rate, $\mathrm {logit}(\mathcal {N})$, in $\hbox {L h}^{-1}$				
$\mathrm {GFR}_\text {rat,young}$	0.0786	Fixed	0.894	20
$\mathrm {GFR}_\text {rat,old}$	0.127	Fixed		
$\mathrm {GFR}_\text {human,male}$	6.92	Fixed	0.381	24
$\mathrm {GFR}_\text {human,female}$	6.045	Fixed		
Blood-to-plasma concentration ratio, dimensionless				
$\mathrm {BP}$	$(1-\mathrm {Hct})$	Fixed	0	Fixed

Reported values refer to the population estimate of the fixed effects on the original (non-transformed) scale, including relative standard error (R.S.E.) of the estimated parameter values

Where appropriate, inter-individual variability was quantified as standard deviation of the random effects on the transformed scale and was denoted by $\omega$. We scaled the GFR fixed-effect parameter as described in the “Methods” section, while the related variability parameter $\omega$ was estimated. Since the transition from $\mathrm {Chl}$ to $\mathrm {Mix}$ was assumed instantaneous, values for the parameters $k_\mathrm {{Chl}2{Mix}}$ and $\mathrm {fu}_\mathrm {Chl}$ from Eqs. (8) and (13) are not required. The assumed values for $\mathrm {Hct}$ can be found in Table 1

### External model validation

**Fig. 6:**
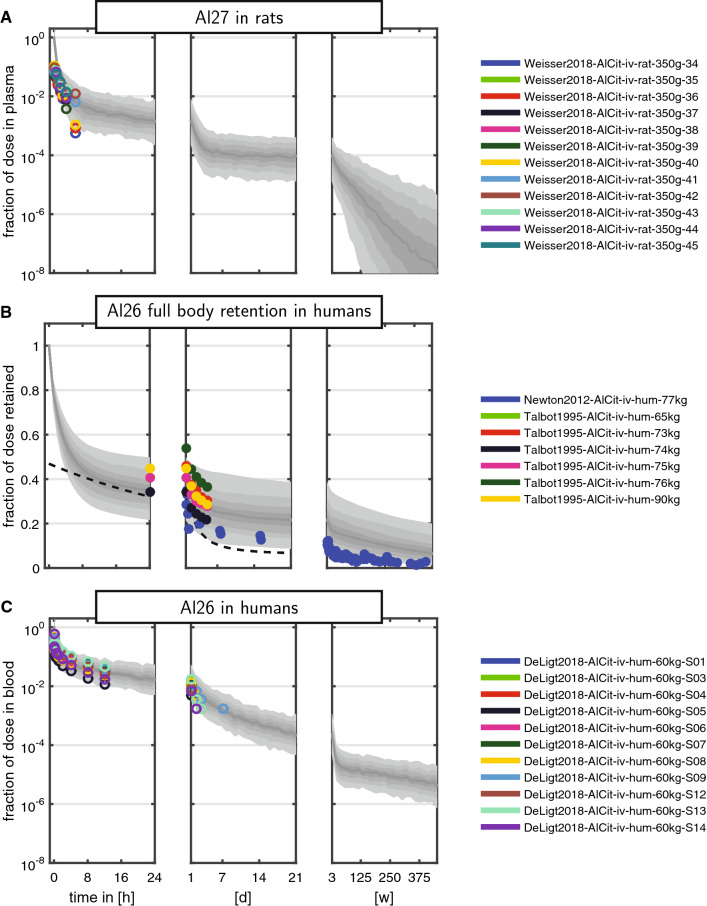
Model validation in rats and humans after single-dose exposure. Colours link to the legend, where the identifier and measured tissues and body fluids are summarised. The shaded areas are the median and the central 20$^{\text {th}}$, 40$^{\text {th}}$, 60$^{\text {th}}$ and 80$^{\text {th}}$ percentiles of the population predictions (based on 250 Monte Carlo simulations). The three panels **a**, **b**, **c** correspond to the three validation datasets described in section “External model validation”. The dashed line in B corresponds to the empirical retention function from Priest (2004); see Eq. (16) (colour figure online)

The results on the first validation dataset (^27^Al in rats) are shown in Fig. 6A. Since no ^26^Al tracer was used, the effective observation time is limited to approximately 4 h. Compared to the observations, the model predicted the kinetics well, including variability.

For the second validation dataset (^26^Al full-body retention in humans), the results are shown in Fig. 6B. The predicted full-body retention matched the observed data and between-subject variability. Of note, since full-body retention data were not part of the training data, no estimate for the residual error of full-body retention was available, and therefore, the shown prediction interval does not include contributions of residual errors. We additionally plotted the empirical retention function from Priest (2004)

17 $$\begin{aligned} \mathrm {ret}(t) = 29 e^{-0.595 t} +11.4 e^{-0.172 t} + 6.5 e^{-0.000401 t}, \end{aligned}$$

with time *t* in days. Based on our simulations, we inferred that 60% of the dose is excreted within the first day, while ‘the next’ 20% is excreted only after about 3 weeks. The ‘final’ 20% are not excreted on the time-scale of any of the observed experiments and a minor fraction may be retained in the body over the entire lifespan.

The results for the third validation dataset (^26^Al in humans) are shown in Fig. 6C. Again, model predictions compared favourably against the observations, including variability.

### Insights into Al toxicokinetics based on the PBTK model

The calibrated model was used to predict and study rat (younger and older adults) and human (male and female) tissues kinetics after administration of aqueous solutions of Al chloride or citrate salts.

**Table 4:** Tissue half-lives of Al in reference humans and rats (see Table 1) based on the final model parameters, computed according to Eq. (18)

	Human		Rat	
Tissue	Male	Female	Young	Old
Muscle	35 h	33 h	5 h	7 h
Kidney	6 days	7 days	4 days	4 days
Spleen	20 days	19 days	8 days	9 days
Liver	28 days	22 days	16 days	18 days
Bone	198 weeks	162 weeks	11 weeks	13 weeks
Brain	$\inf$	$\inf$	$\inf$	$\inf$

‘Young’ and ‘Old’ refer to adult rats with body weight of 250 g and 480 g, respectively

Release of Al from tissues is characterised by the tissue release rate constants $k_\mathrm {{\mathrm {tis}}2{\mathrm {blo}}}$ defined in Eq. (6), yielding the tissue half-life

18 $$\begin{aligned} t_\text {1/2} = \frac{\log (2)}{k_\mathrm {{\mathrm {tis}}2{\mathrm {blo}}}} = \frac{\log (2)\times K_\mathrm {tis}V_\mathrm {tis}}{Q_\mathrm {tis}}. \end{aligned}$$

Table 4 reports the different tissue half-lives for the four reference individuals/species. In general, three groups of tissues with respect to half-life were identified: (i) quickly releasing tissues (including muscle) with $t_{1/2}$ on the time-scale of hours; (ii) intermediately releasing tissues (including liver, spleen and kidney) with $t_{1/2}$ on the time-scale of days; and (iii) slowly/not releasing tissues (including bone and brain) with $t_{1/2}$ on the time-scale of weeks. Differences in physiology between rat and human, reflected in different ratio of blood flows to organ volumes (see Table 1), determine the inter-species differences in $t_{1/2}$. Overall, humans have longer half-lives in all tissues, with the most pronounced differences in muscle and bone.

**Fig. 7:**
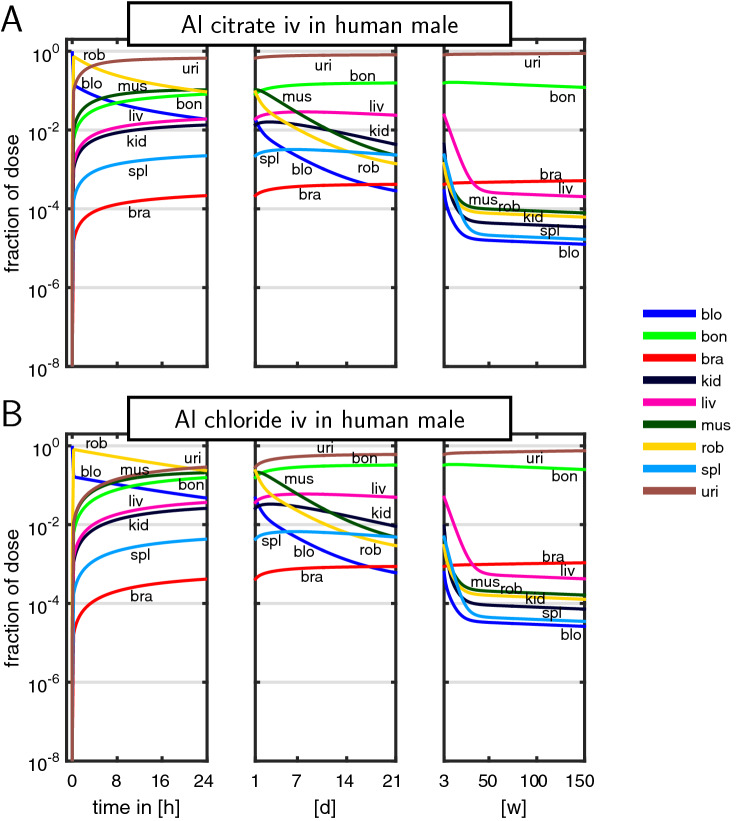
Disposition pattern (fraction of ingested dose) in the human male reference individual after single iv administration of aqueous solutions of Al citrate (**a**) and chloride (**b**) (colour figure online)

Figure 7 shows the time course of the fraction of ingested dose (predicted mean) after a single iv administration of Al citrate and Al chloride in man. In both cases, Al in blood decays very quickly within 24 h to 2% and 5%, respectively. At the same time, Al redistributes in the ‘rest of body’ and the tissue compartments. While the amount of Al in all other compartments increases during the first 24 h, plasma and rest of body decay in parallel after having reached their maximum fid value. Over the time course of 150 weeks, for Al citrate, about 87% is excreted in urine; bone peaks at approx. 16%, while brain continuously increases up to 0.05%. For Al chloride, elimination is slower, so about 75% is excreted in urine, while Al accumulates more strongly in bone and brain: bone peaks at approx. 33%, while brain continuously increases up to 0.1%.

## Discussion

We presented a novel toxicokinetic model for iv and po single-dose Al exposure that is physiology-based and proved to be capable of describing the most comprehensive set of ^26^Al study data available today in rats and humans after intravenous and oral single-dose administration. Key features of our model include (i) known aspects of Al speciation; (ii) consideration of physiologically or toxicologically relevant tissues; (iii) differences in Al kinetics between rat and human as a result of differences in species physiology; (iv) integration into a statistical framework to adequately quantify uncertainty; (v) validation for single-dose exposure in rats and humans (adults). Due to its inter-species extrapolation capabilities, the PBTK model allowed translating tissue distribution data from rat studies to the human situation, while at the same time accounting for species differences. Compared to prior models that have been reviewed and discussed in detail in Weisser et al. (2017), this constitutes a major step forward in our understanding of Al TK, in particular in humans. It thus constitutes a key advancement in a field that controversially discusses toxic effects related to acute and chronic exposure to Al without having a validated model for prediction purposes.

As a key prerequisite, we compiled a unique comprehensive and curated dataset of ^26^Al data in rats and humans after iv and po administration of aqueous solutions of Al chloride and citrate salts. This dataset allows to analyse, for the first time, ^26^Al kinetics beyond particular features of individual studies. The most frequently encountered problems revealed during the curation process were incomplete information on the experiments and duplicates. In terms of harmonisation, the use of PB scaling methods enabled the consolidation of units to the common unit ‘fraction of ingested dose’. The PB scaling method, however, may also introduce additional uncertainty due to unknown individual organ weights. Of note, reported measurements are also often subject to scaling, e.g., when samples from liver, muscle, bone, and spleen are scaled up to the whole organ. With respect to minimisation of the impact of uncertainty introduced by up-scaling and unit conversions, we realised the importance of transparency by experimentalists in reporting organ weights used for scaling.

The outlined de-aggregation of summarised data ensured to obtain samples with the originally reported mean and standard deviation; see Eq. (2). This sampling introduces randomness in the process of dataset generation. To assess, whether a statistical analysis of these data, e.g., model calibration, is sensitive on this process, we suggest (as we did) to iteratively generate and analyse such datasets to compare the results.

Despite its richness, the comprehensive curated dataset still has ‘blind spots’. It is of limited diversity with respect to sex and age: almost all studies in rats and humans were performed in males. From the references in the curated dataset, only Moore et al. (1997) includes a human female participant (Moore1997-AlCit-po-hum-60kg). Age was not regularly reported numerically in the original studies. For humans, only adults were studied. A similar statement holds in rats when BW is used as proxy for age (Sengupta 2013). Only in Zafar et al. (1997), the authors performed a study in pubescent rats. Regarding the sampling matrix, reported data in humans are restricted to blood, plasma, and urine due to obvious ethical reasons. In rats, data in a variety of tissues are available. With one exception, the available ^26^Al study data are confined to single dosing. Only in Steinhausen et al. (2004), the human 79 kg BW individual was dosed a second time after 2 years. While in humans, several iv studies with both Al salts (citrate and chloride) are available, for rats only one study administered ^26^Al intravenously as citrate salt (Yokel et al. 2001b). Non-linear clearance is reported for *intraveneous* doses above 3E–5 mol Al/kg BW in rats (see Wilhelm et al. 1992). Since all doses are below this threshold (iv doses in rats ranged from 2E–8 to 3E–6 mol Al/kg BW, see Table 2), such effects are likely not present in the curated dataset and were considered negligible.

We observed overall high levels of inter-study and inter-individual variability. Such levels are expected due to the lack of standardised study protocols, even in light of the precise measurements of the ^26^Al content in the samples. At the same time, within-study standardisation has been identified as a cause of poor reproducibility (Voelkl et al. 2018). Thus, the compilation of a rich dataset from diverse studies may increase the robustness on any deduced findings.

Using the curated data set, model parameters were estimated with good precision and resulted in convincing goodness-of-fit, in particular in the light of high variability and non-standardised experimental protocols. Specifically, the PBTK model was able to accurately reproduce species differences (rat and human) and the impact of the routes of administration (iv and po) and Al salts (citrate and chloride). Furthermore, the estimated parameters were robust to changes in the initialisation of the estimation algorithm and randomness during generation of the training dataset. For all three validation datasets, our model precisely predicted the corresponding time-profiles, even for long time spans of up to 375 weeks (Newton and Talbot 2012). While the retention dataset itself was not part of the training data, the same individuals, however, were included in the training dataset with plasma, blood, and urine samples. Since these observations, especially the urine samples, could inform the full-body retention kinetics, the full-body retention data should not be considered a full external validation, but rather somewhere between an internal and external validation.

The species differences between rat and human that we accounted for in the PBTK model were blood flows, organ volumes, GFR, and Hct. All other differences were subsumed under unexplained variability and parameter uncertainty. Additional known and possibly Al-related physiological characteristics differentiate rats from humans and may be incorporated in the future, including differences in muscles (more fast-twitch fibres) and bones (no Haversian remodelling (Bentolila et al. 1998; Lelovas et al. 2008) as well as a faster bone turnover (Manolagas 2000; Sontag 1986)). Currently, however, inclusion of further mechanistic details is limited by sparsity of data and knowledge, resulting in parameter identifiability problems.

In the present PBTK model, the ‘rest of body’ compartment accounts for some of the unknown processes of Al kinetics—in addition to parameter uncertainty and the residual error model. The disposition patterns in Fig. 7 clearly show a knowledge gap during the first 24 h, during which the ‘rest of body’ compartment is associated with the largest fid. Removing the ‘rest of body’ compartment from the model reduced the goodness-of-fit in corresponding plots, indicating the presence of some additional distributional space that might be associated with carcass, adipose tissue, and lung or sites escaping quantification of tissue homogenates of the considered tissues. As Nolte et al. (2001) already suggested, a fast distribution of hydrophilic Al salts into the total extracellular body water (including interstitial fluid) is plausible. This would include the interstitial space of adipose and lung tissue being part of our rob compartment, and would suggest a parallel decay of plasma and rest of body (as was observed in Fig. 7). Furthermore, due to lack of specific knowledge and data, we assumed a well-stirred tissue model, i.e., a fast exchange across distributional barriers between vascular, interstitial, and cellular space. For the same reason, we were not able to account for possible differences in active uptake and release transports (e.g., depending on the Al species). All this might additionally contribute to the ‘rest of body’ compartment. Importantly, on the time-scale of weeks and month, the impact of the ‘rest of body’ compartment is negligible (see Fig. 7).

With respect to the complex and only partially understood Al speciation in vivo (Michalke et al. 2009), our PBTK model includes some aspects only: (i) the difference in renal filterability of Al citrate compared to Al chloride (Shirley and Lote 2005), which implies a higher plasma clearance for AlCit compared to other salts and was confirmed in our recent experiments after iv injection of Al citrate in rats (Weisser et al. 2019); (ii) the necessary process of equilibration between all binding partners of Al in blood (mainly citrate and transferrin) reflected in the term $k_\mathrm {{\mathrm {Cit}}2{\mathrm {Mix}}}$. This allows fast renal elimination of Al citrate before quasi-equilibrium between all competing binding partners of Al in blood is reached ($A_\mathrm {Mix}$). It follows that from time of administration up to equilibration, AlCit and AlChl differ in clearance for iv application. As equilibration is reached within 24 h (see estimate for $k_\mathrm {{\mathrm {Cit}}2{\mathrm {Mix}}}$ in Table 3), the overall impact on the kinetics is rather low. A more detailed (mechanistic) model of Al speciation is conceivable, but not supported by our ^26^Al data basis. For example, in certain scenarios of Al exposure, especially involving colloidal or non-soluble states of Al, non-convective distribution may alter the kinetics. It is hypothesised that the reticulo-endothelial system represents an additional uptake pathway for insoluble colloids, thereby allowing Al to escape the highly effective renal elimination from blood (Priest 2004; Krewski et al. 2007). Thus, the extension of the model to simulate exposure to other relevant but potentially less soluble aluminium species like some hydroxides, oxides, hydroxide oxides, and silicates is complex and needs modifications of the absorption model including potential dynamic interconversion rates between the species. To this end, further experimental plasma and tissue data are a prerequisite.

The PBTK model predicted tissue accumulation of different degree; tissues with long half-life (bone and brain) are predicted to accumulate Al during continuous exposure. Only a small number of samples for these tissues (11/162), however, were beyond 21 days of observation, which resulted in an increased uncertainty for these tissue distribution parameters. The conservative modelling of brain as a sink compartment has a strong impact on predicted brain tissue distribution on the time-scale of decades.

The presented PBTK model was calibrated and validated on *single-dose* Al data only. Of course, there is regulatory interest in application of the model for simulations of repeated administrations of Al compounds (e.g., from medicinal products such as antacids, vaccines or subcutaneous immunotherapeutics) or even continuous long-term exposure of Al (e.g., occupational or dietary exposure). In this regard, human biomonitoring data as well as autopsy data may prove valuable to inform the long-term within body distribution in humans. Using the present PBTK model for multiple dosing would implicitly assume that no additional processes, like saturation, etc., would come into play after multiple dosing. For simulation of live-long exposure scenarios, the PBTK model would also need to include maturation effects during childhood and age-related changes in the elderly (e.g., bone homeostasis and GFR). Although this is beyond the scope of this article, we nevertheless simulated continuous dietary exposure to an amount of Al corresponding to the TWI. We found the resulting blood concentrations for long-term exposure to be in the same order of magnitude as average levels reported for healthy humans (Krewski et al. 2007). This may indicate that our model might be usable—with possible modifications related to the input compartment—for simulations of various exposure scenarios in the future.

We made the assumptions of the clearance being exclusively renal, as Priest (2004) reported only $\approx 1\%$ of an iv dose of Al citrate being excreted via faeces. Based on the goodness-of-fit of our model, there was no indication that non-renal clearance contributed in a relevant manner to overall Al kinetic after iv and po administration. However, routes of excretion other than urine and faeces are described, e.g., via sweat, skin, hair, nails, sebum, and semen (Exley 2013), but on the basis of all iv and po ^26^Al data, we conclude that they are quantitatively irrelevant.

Children and infants represent an important special population of key interest for risk assessment. It is important to stress that they age-dependently show remarkable differences in bone metabolism compared to the reference adult individuals used in the present study. As overall Al kinetics are likely to be very sensitive to altered bone uptake and release, altered bone maturation processes are likely to play a similarly important role. Consequently, to extend the presented model to children and infants, implementation of a refined bone model should be considered.

In conclusion, based on the model development process, the goodness-of-fit, and the validation, we are confident that our PBTK model is suitable for studying the internal Al exposure after a single dose of intravenously and per orally administered aqueous solution of Al citrate and Al chloride in rats and humans. The use of the model for multiple or continuous administration of Al would need further model validation or should be accompanied with a critical discussion on the associated uncertainties and their implications. The same applies to extrapolation to specific subgroups, such as newborn, neonates, children, and the elderly. To this end, the use of the large pool of ^27^Al data in the literature might be beneficial, although on the other hand, baseline levels and contamination constitute serious limitations.

The systemic PBTK model can also serve as a starting point to include other routes of exposure, like dermal administration or inhalation. Concerning the dermal route, currently 2 published studies build the body of evidence for ^26^Al: Flarend et al. (2001), de Ligt et al. (2018). However, the development of a purely data-driven dermal absorption model may be impaired by the small sample size and large number of censored observations. The exposure via inhalation is in our opinion currently out of reach due to missing ^26^Al kinetic data.

In summary, the presented PBTK model for Al constitutes a major advancement as it has been build on the most extensive and diverse dataset of intravenously and per orally administered Al exposure to date, thereby paving the way towards a more quantitative risk assessment in humans. Specifically, model extensions to simulate risks associated with Al containing medications, vaccinations, or cosmetics administered to humans at all age groups are now within reach.

## Supplementary Information

Below is the link to the electronic supplementary material.

Supplementary material 1 (pdf 235 KB)

Supplementary material 2 (7z 1504 KB)

## Data Availability

All data, material, and code are available in the electronic supplement.
