# Supplementary material for: Physiology-based toxicokinetic modelling of aluminium in rat and man
Source: Arch Toxicol. 2021 Aug 14;95(9):2977–3000. doi: 10.1007/s00204-021-03107-y (PMC8380244; doi:10.1007/s00204-021-03107-y)
Supplement: Supplementary file 1 — Supplementary material 1 (pdf 235 KB) [file 204_2021_3107_MOESM1_ESM.pdf]

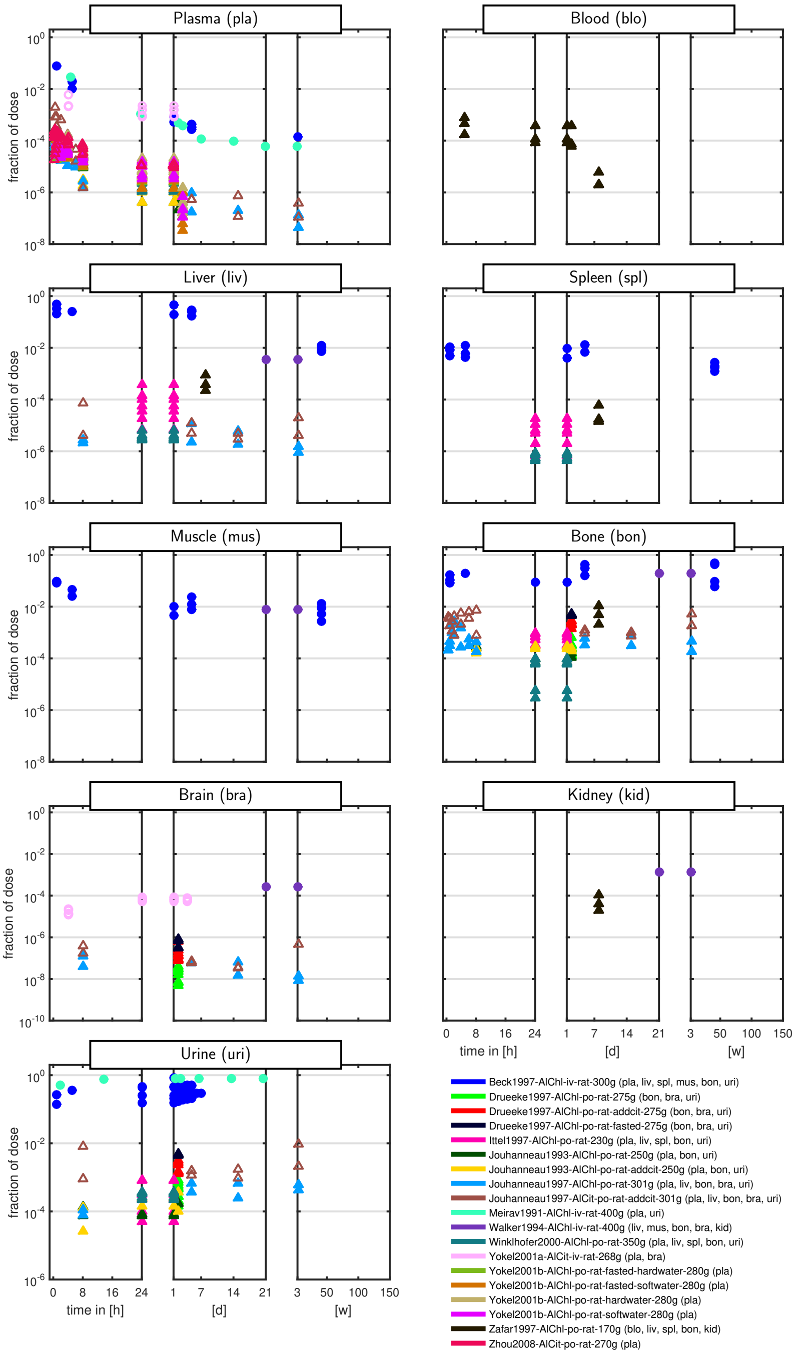

Fig. S1 Al disposition in rats after single iv (circles) or po (triangles) administration of aqueous solutions containing Al citrate (filled symbols) and Al chloride (open symbols). Note the different time-scales on the three panels: hours (h), days (d) and weeks (w).

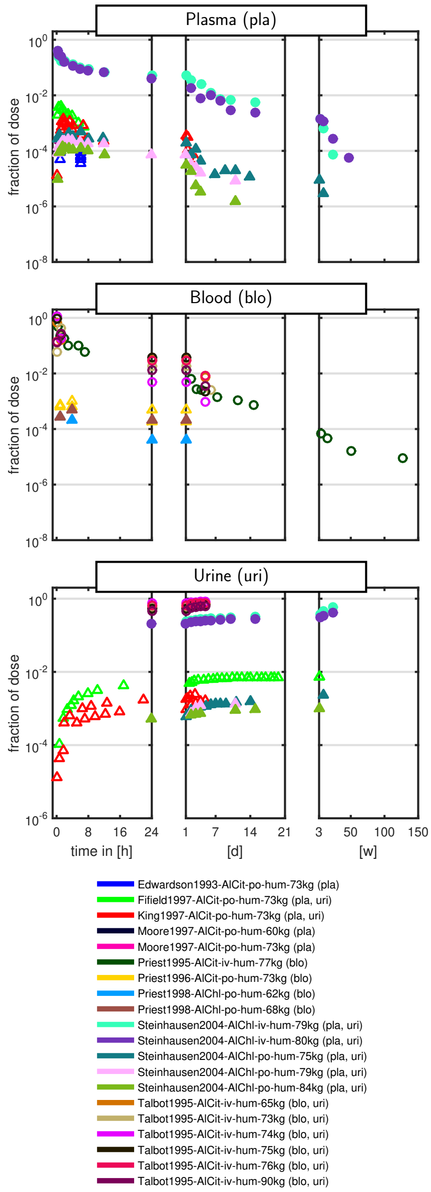

Fig. S2 Al disposition in humans after single iv (circles) or po (triangles) administration of aqueous solutions containing Al citrate (filled symbols) and Al chloride (open symbols). Note the different time-scales on the three panels: hours (h), days (d) and weeks (w).

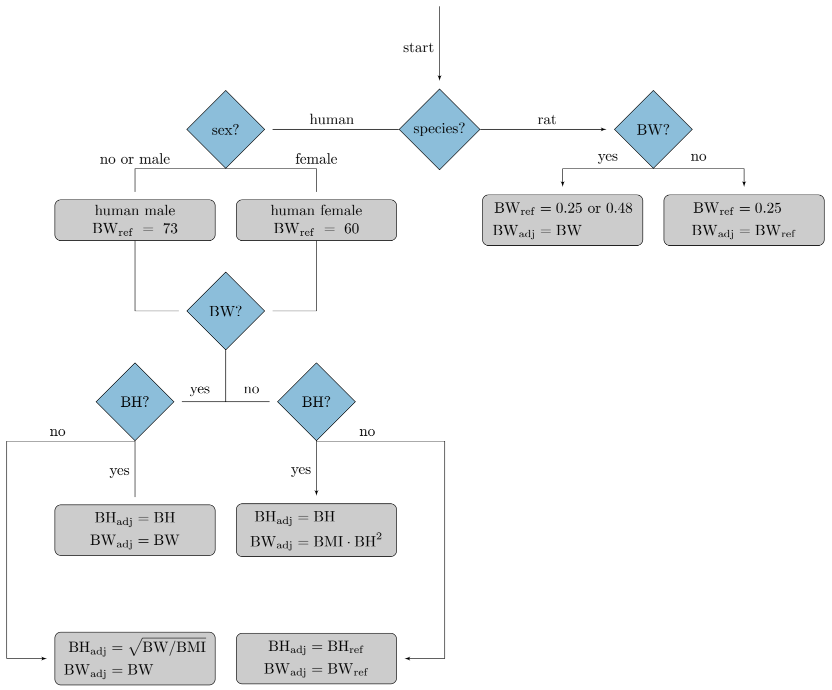

Fig. S3 Decision tree for missing data. For each identifier, physiological parameters were scaled based on reference individuals defined in the methods section of the main article. Questions refer to whether the variable was reported in the study or not. Young (0.25 kg) or old (0.48 kg) reference rats are chosen based on whichever is closest to the adjusted body weight (BW). For reference humans, additionally sex and body height (BH) determine this choice. If BW, BH and/or sex is not reported in the original study, imputation for these variables followed the depicted decision tree. For the human body mass index we assumed $BMI = 25\,kg/m^2$.

Table S1 References excluded from the curated dataset (human). Abbreviations: Duplicate (DUP), not included tissue (TIS), reserved for validation (VAL), not included chemical species (CHE), not included administration (ADM), not included unit (UNI), implausible time point $t < 1\,\mathrm{s}$ (TIM), not included health status (HEA), implausible value $y \leq 0$ (CEN), not included special treatment (SPE), no reported variance (VAR). For tissue abbreviations see main article. While excluded from the curated dataset, these observations are part of the comprehensive dataset and may be inspected in the supplemental material.

Reference	Number of excluded samples and comment
(Day et al., 1991)	1 human AlCit po sample (pla)<br>due to DUP (Edwardson et al., 1993)
(de Ligt et al., 2018)	130 human AlCit iv samples (blo)<br>due to VAL
(de Ligt et al., 2018)	77 human AlCit iv samples (spot uri)<br>due to VAL, TIS
(Flarend et al., 2001)	6 human AlCit dermal samples (pla)<br>due to CHE (Al-Chlorohydrate), ADM,<br>UNI (Fraction of applied Al / g)
(King et al., 1997)	2 human AlCit po samples (pla, uri)<br>due to TIM
(King et al., 1997)	1 human AlCit po sample (uri)<br>due to CEN
(King et al., 1997)	55 human AlCit po samples (pla-uri)<br>due to SPE (added high silicate)
(King et al., 1997)	2 human AlCit po samples (pla-uri)<br>due to SPE (added high silicate), TIM
(Moore et al., 1997)	5 human AlCit po samples (pla)<br>due to HEA (tris21)
(Moore et al., 2000)	13 human AlCit po samples (pla)<br>due to UNI (relative uptake factor)
(Moore et al., 2000)	13 human AlCit po samples (pla)<br>due to HEA (Alzheimers), UNI (relative uptake factor)
(Nolte et al., 2001)	25 human AlCit iv samples (pla-uri)<br>due to DUP (Priest et al., 1995)
(Nolte et al., 2001)	8 human AlCit iv samples (faeces)<br>due to DUP (Priest et al., 1995), TIS
(Priest et al., 1996)	2 human AlCit po samples (blo)<br>due to TIM
(Priest et al., 1996)	2 human AlHyd with AlCit po samples (blo)<br>due to CHE (Al-Hydroxide), TIM
(Priest et al., 1996)	6 human AlHyd po samples (blo)<br>due to CHE (Al-Hydroxide)
(Priest et al., 1996)	2 human AlHyd with AlCit samples (blo)<br>due to CHE (Al-Hydroxide), TIM
(Priest et al., 1996)	6 human AlHyd with AlCit po samples (blo)<br>due to CHE (Al-Hydroxide)
(Priest et al., 1998)	2 human AlChl po samples (blo)<br>due to TIM
(Steinhausen et al., 2004)	57 human AlChl po samples (pla-uri)<br>due to HEA (chronic glomerulonephritis)

Table S2 References excluded from the curated dataset (rats). Abbreviations: Duplicate (DUP), not included tissue (TIS), reserved for validation (VAL), not included chemical species (CHE), not included administration (ADM), not included unit (UNI), implausible time point $t < 1\,\mathrm{s}$ (TIM), not included health status (HEA), implausible value $y \leq 0$ (CEN), not included special treatment (SPE), no reported variance (VAR). For tissue abbreviations see main article. While excluded from the curated dataset, these observations are part of the comprehensive dataset and may be inspected in the supplemental material.

Reference	Number of excluded samples and comment
(Beck, 1997)	58 rat AlChl iv samples (uri-serum-liv-spl-mus-bon) due to SPE (nephretomized)
(Drueeke et al., 1997)	16 rat AlChl po samples (bon-uri) due to SPE (added high silicate)
(Drueeke et al., 1997)	16 rat AlChl po samples (bon-uri) due to SPE (added high silicate and citrate)
(Fink et al., 1994)	5 rat po samples (bra-greymatter-liv) due to CHE (unkown)
(Ittel et al., 1997)	30 rat AlChl po samples (serum-liv-bon-spl-uri) due to SPE (nephretomized)
(Kobayashi et al., 1990)	15 rat AlChl ip samples (liv-bra-blo) due to ADM
(Meirav et al., 1990)	14 rat AlChl iv samples (serum-uri) due to DUP (Meirav et al., 1991)
(Meirav et al., 1991)	14 rat AlChl iv samples (serum-uri) due to SPE (nephretomized)
(Schoenholzer et al., 1997)	6 rat AlCit po samples (pla-uri) due to VAR
(Schoenholzer et al., 1997)	7 rat AlCit with AlCit po samples (pla-uri) due to VAR
(Schoenholzer et al., 1997)	7 rat AlHyd po samples (pla-uri) due to VAR, CHE (Al-Hydroxide)
(Schoenholzer et al., 1997)	7 rat AlMal po samples (pla-uri) due to VAR, CHE (Al-Maltotate)
(Steinhausen et al., 1996)	12 rat AlChl iv samples (serum) due to DUP (Beck, 1997; Steinhausen et al., 2004)
(Steinhausen, 1997)	34 rat AlChl iv samples (serum-bon-uri-liv-spl-mus) due to DUP (Beck, 1997; Steinhausen et al., 2004)
(Steinhausen, 1997)	27 rat AlChl iv samples (serum-bon-uri-liv-spl-mus) due to DUP (Beck, 1997; Steinhausen et al., 2004), SPE (nephrectomized)
(Steinhausen, 1997)	5 rat AlChl po samples (serum-uri-liv-spl-bon) due to DUP (Beck, 1997; Steinhausen et al., 2004)
(Steinhausen, 1997)	5 rat AlChl po samples (serum-uri-liv-spl-bon) due to DUP (Winklhofer et al., 2000)
(Steinhausen, 1997)	5 rat AlChl po samples (serum-uri-liv-spl-bon) due to DUP (Winklhofer et al., 2000), SPE (iron deficient diet)
(Steinhausen, 1997)	5 rat AlChl po samples (serum-uri-liv-spl-bon) due to DUP (Winklhofer et al., 2000), SPE (iron saturated diet)
(Steinhausen, 1997)	5 rat AlChl po samples (serum-uri-liv-spl-bon) due to DUP (Beck, 1997; Steinhausen et al., 2004), SPE (nephretomized)

Table S3 References excluded from the curated dataset (rats); continuation of Table S2. Abbreviations: Duplicate (DUP), not included tissue (TIS), reserved for validation (VAL), not included chemical species (CHE), not included administration (ADM), not included unit (UNI), implausible time point $t < 1\,\mathrm{s}$ (TIM), not included health status (HEA), implausible value $y \leq 0$ (CEN), not included special treatment (SPE), no reported variance (VAR). For tissue abbreviations see main article. While excluded from the curated dataset, these observations are part of the comprehensive dataset and may be inspected in the supplemental material.

Reference	Number of excluded samples and comment
(Walker and Sutton, 1994)	1 rat AlChl iv sample (heart)<br>due to TIS
(Walker and Sutton, 1994)	5 rat AlChl iv samples (bon-kid-liv-bra-mus)<br>due to SPE (nephretomized)
(Walker and Sutton, 1994)	1 rat AlChl iv sample (heart)<br>due to SPE (nephretomized), TIS
(Walton et al., 1995)	8 rat po samples (bra)<br>due to CHE (unknown)
(Winklhofer et al., 2000)	48 rat AlChl po samples (uri-bon-spl-liv-pla)<br>due to SPE (iron deficient diet)
(Winklhofer et al., 2000)	44 rat AlChl po samples (uri-bon-spl-liv-pla)<br>due to SPE (iron saturated diet)
(Yokel et al., 2001b)	60 rat AlTf iv samples (bra-serum)<br>due to CHE (Al-Transferrin)
(Yokel et al., 2001b)	30 rat AlTf iv samples (bra)<br>due to CHE (Al-Transferrin), SPE (added desferriox-amine)
(Yokel and Florence, 2006)	16 rat AlHyd po samples (serum)<br>due to CHE (Al-Hydroxide)
(Yokel et al., 2008)	16 rat AlSal po samples (serum)<br>due to CHE (Al-sodium-phosphate)
(Yokel and Florence, 2008)	8 rat AlCit po samples (serum)<br>due to VAR
(Yokel and Florence, 2008)	8 rat AlTea po samples (serum)<br>due to no number of replicates, CHE (AlCit in tea)
(Yumoto et al., 1997)	24 rat AlChl ip samples (blo-bra-liv)<br>due to ADM (intraperitoneal)
(Yumoto et al., 2000)	9 rat AlChl lac samples (kid-liv-bra-blo)<br>due to ADM (lactation)
(Yumoto et al., 2000)	1 rat AlChl sc sample (liv)<br>due to ADM (subcutanous)
(Yumoto et al., 2000)	2 rat AlChl sc samples (placenta-fetuses)<br>due to ADM (subcutanous), TIS
(Zafar et al., 1997)	24 rat AlChl ip samples (spl-liv-kid-bon-blo)<br>due to ADM (intraperitoneal)
(Zhou et al., 2008)	49 rat AlMal po samples (serum)<br>due to CHE (Al-Maltotate)

References

Beck E (1997) Langzeituntersuchung der Aluminiumkinetik mit Beschleuniger-massenspektrometrie. Diploma thesis, Technischen Universität München

Day JP, Barker J, Evans LJ, Perks J, Seabright PJ, Ackrill P, Lilley JS, Drumm PV, Newton GW (1991) Aluminum absorption studied by 26Al tracer. Lancet (London, England) 337(8753):1345, DOI 10.1016/0140-6736(91)93016-3

de Ligt R, van Duijn E, Grossouw D, Bosgra S, Burggraaf J, Windhorst A, Peeters PA, van der Luijt GA, Alexander-White C, Vaes WH (2018) Assessment of Dermal Absorption of Aluminum from a Representative Antiperspirant Formulation Using a 26Al Microtracer Approach. Clinical and Translational Science DOI 10.1111/cts.12579

Drueeke TB, Jouhanneau P, Banide H, Lacour B, Yiou F, Raisbeck G (1997) Effects of silicon, citrate and the fasting state on the intestinal absorption of aluminium in rats. Clinical Science (London, England: 1979) 92(1):63–67

Edwardson J, Moore P, Ferrier I, Lilley J, Barker J, Templar J, Day J (1993) Effect of silicon on gastrointestinal absorption of aluminium. The Lancet 342(8865):211–212, DOI 10.1016/0140-6736(93)92301-9

Fink D, Walton J, Hotchkis MAC, Jacobsen GE, Lawson EM, Smith AM, Tuniz C, Wilcox D (1994) First 26Al analyses at the ANTARES AMS Centre: Uptake via oral ingestion of 26Al in rats. Nuclear Instruments and Methods in Physics Research Section B: Beam Interactions with Materials and Atoms 92(1):473–477, DOI 10.1016/0168-583X(94)96057-7

Flarend R, Bin T, Elmore D, Hem S (2001) A preliminary study of the dermal absorption of aluminium from antiperspirants using aluminium-26. Food and Chemical Toxicology 39(2):163–168, DOI 10.1016/S0278-6915(00)00118-6

Ittel TH, Steinhausen C, Kislinger G, Kinzel S, Nolte E, Sieberth HG (1997) Ultrasensitive analysis of the intestinal absorption and compartmentalization of aluminium in uraemic rats: A 26Al tracer study employing accelerator mass spectrometry. Nephrology Dialysis Transplantation 12(7):1369–1375, DOI 10.1093/ndt/12.7.1369

King SJ, Day JP, Oldham C, Popplewell JF, Ackrill P, Moore PB, Taylor GA, Edwardson JA, Fifield LK, Liu K, Cresswell RG (1997) The influence of dissolved silicate on the physiological chemistry of aluminium, studied in humans using tracer 26Al and accelerator mass spectrometry. Nuclear Instruments and Methods in Physics Research Section B: Beam Interactions with Materials and Atoms 123(1):254–258, DOI 10.1016/S0168-583X(96)00699-4

Kobayashi K, Yumoto S, Nagai H, Hosoyama Y, Imamura M, Masuzawa Si, Koizumi Y, Yamashita H (1990) 26Al tracer experiment by accelerator mass spectrometry and its application to the studies for amyotrophic lateral sclerosis and Alzheimer's disease. I. Proceedings of the Japan Academy 66(10):189–192

Meirav O, Sutton R, Fink D, Middleton R, Klein J, Walker V, Halabe A, Vetterli D, Johnson R (1990) Application of accelerator mass spectrometry in aluminum metabolism studies. Nuclear Instruments and Methods in

Physics Research Section B: Beam Interactions with Materials and Atoms 52(3-4):536–539, DOI 10.1016/0168-583X(90)90472-7

Meirav O, Sutton RA, Fink D, Middleton R, Klein J, Walker VR, Halabe A, Vetterli D, Johnson RR (1991) Accelerator mass spectrometry: Application to study of aluminum kinetics in the rat. American Journal of Physiology-Renal Physiology 260(3):F466–F469, DOI 10.1152/ajprenal.1991.260.3.F466

Moore PB, Edwardson JA, Ferrier IN, Taylor GA, Lett D, Tyrer SP, Day JP, King SJ, Lilley JS (1997) Gastrointestinal absorption of aluminum is increased in down's syndrome. Biological Psychiatry 41(4):488–492, DOI 10.1016/S0006-3223(96)00045-5

Moore PB, Day JP, Taylor GA, Ferrier IN, Fifield LK, Edwardson JA (2000) Absorption of Aluminium-26 in Alzheimer's Disease, Measured Using Accelerator Mass Spectrometry. Dementia and Geriatric Cognitive Disorders 11(2):66–69, DOI 10.1159/000017216

Nolte E, Beck E, Winklhofer C, Steinhausen C (2001) Compartmental model for aluminium biokinetics. Human & Experimental Toxicology 20(2):111–117, DOI 10.1191/096032701673730925

Priest N, Newton D, Day J, Talbot R, Warner A (1995) Human metabolism of aluminium-26 and gallium-67 injected as citrates. Human & Experimental Toxicology 14(3):287–293, DOI 10.1177/096032719501400309

Priest ND, Talbot RJ, Austin JG, Day JP, King SJ, Fifield K, Cresswell RG (1996) The bioavailability of 26Al-labelled aluminium citrate and aluminium hydroxide in volunteers. BioMetals 9(3):221–228, DOI 10.1007/BF00817919

Priest ND, Talbot RJ, Newton D, Day JP, King SJ, Fifield LK (1998) Uptake by man of aluminium in a public water supply. Human & Experimental Toxicology 17(6):296–301, DOI 10.1177/096032719801700602

Schoenholzer KW, Sutton RAL, Walker VR, Sossi V, Schulzer M, Orvig C, Venczel E, Johnson RR, Vetterli D, Dittrich-Hannen B, Kubik P, Suter M (1997) Intestinal Absorption of Trace Amounts of Aluminium in Rats Studied with 26 Aluminium and Accelerator Mass Spectrometry. Clinical Science 92(4):379–383, DOI 10.1042/cs0920379

Steinhausen C (1997) Untersuchung der Aluminiumbiokinetik mit 26Al und Beschleunigermassenspektrometrie. Dissertation, Technische Universität München

Steinhausen C, Gerisch P, Heisinger B, Hohl C, Kislinger G, Korschinek G, Niedermayer M, Nolte E, Dumitru M, Alvarez-Brückmann M, Schneider M, Ittel TH (1996) Medical application of 26Al. Nuclear Instruments and Methods in Physics Research Section B: Beam Interactions with Materials and Atoms 113(1):479–483, DOI 10.1016/0168-583X(95)01378-4

Steinhausen C, Kislinger G, Winklhofer C, Beck E, Hohl C, Nolte E, Ittel TH, Alvarez-Brückmann MJ (2004) Investigation of the aluminium biokinetics in humans: A 26Al tracer study. Food and Chemical Toxicology 42(3):363–371, DOI 10.1016/j.fct.2003.09.010

Walker VR, Sutton RAL (1994) Tissue disposition of 26aluminum in rats measured by accelerator mass spectrometry. Clin Invest Med 17(5):6

Walton J, Tuniz C, Fink D, Jacobsen G, Wilcox D (1995) Uptake of trace amounts of aluminum into the brain from drinking water. Neurotoxicology 16(1):187–190

Winklhofer C, Steinhausen C, Beck E, Alvarez-Brückmann M, Kinzel S, Ittel TH, Nolte E (2000) Effect of iron status on the absorption, speciation and tissue distribution of aluminium in rats. Nuclear Instruments and Methods in Physics Research Section B: Beam Interactions with Materials and Atoms 172(1):920–924, DOI 10.1016/S0168-583X(00)00228-7

Yokel RA, Florence RL (2006) Aluminum bioavailability from the approved food additive leavening agent acidic sodium aluminum phosphate, incorporated into a baked good, is lower than from water. Toxicology 227(1-2):86–93, DOI 10.1016/j.tox.2006.07.014

Yokel RA, Florence RL (2008) Aluminum bioavailability from tea infusion. Food and Chemical Toxicology: An International Journal Published for the British Industrial Biological Research Association 46(12):3659–3663, DOI 10.1016/j.fct.2008.09.041

Yokel RA, Rhineheimer SS, Brauer RD, Sharma P, Elmore D, McNamara PJ (2001b) Aluminum bioavailability from drinking water is very low and is not appreciably influenced by stomach contents or water hardness. Toxicology 161(1-2):93–101, DOI 10.1016/S0300-483X(01)00335-3

Yokel RA, Hicks CL, Florence RL (2008) Aluminum bioavailability from basic sodium aluminum phosphate, an approved food additive emulsifying agent, incorporated in cheese. Food and Chemical Toxicology 46(6):2261–2266, DOI 10.1016/j.fct.2008.03.004

Yumoto S, Nagai H, Imamura M, Matsuzaki H, Hayashi K, Masuda A, Kumazawa H, Ohashi H, Kobayashi K (1997) 26Al uptake and accumulation in the rat brain. Nuclear Instruments and Methods in Physics Research Section B: Beam Interactions with Materials and Atoms 123(1):279–282, DOI 10.1016/S0168-583X(96)00429-6

Yumoto S, Nagai H, Matsuzaki H, Kobayashi T, Tada W, Ohki Y, Kakimi S, Kobayashi K (2000) Transplacental passage of Al from pregnant rats to fetuses and Al transfer through maternal milk to suckling rats. Nuclear Instruments and Methods in Physics Research Section B: Beam Interactions with Materials and Atoms 172(1-4):925–929, DOI 10.1016/S0168-583X(00)00096-3

Zafar TA, Weaver CM, Martin BR, Flarend R, Elmore D (1997) Aluminum (26Al) Metabolism in Rats. Experimental Biology and Medicine 216(1):81–85, DOI 10.3181/00379727-216-44159

Zhou Y, Harris WR, Yokel RA (2008) The influence of citrate, maltolate and fluoride on the gastrointestinal absorption of aluminum at a drinking water-relevant concentration: A 26Al and 14C study. Journal of Inorganic Biochemistry 102(4):798–808, DOI 10.1016/j.jinorgbio.2007.11.019
